# Exploiting *Issatchenkia orientalis* SD108 for succinic acid production

**DOI:** 10.1186/s12934-014-0121-4

**Published:** 2014-08-27

**Authors:** Han Xiao, Zengyi Shao, Yu Jiang, Sudhanshu Dole, Huimin Zhao

**Affiliations:** Department of Chemical and Biomolecular Engineering, University of Illinois at Urbana-Champaign, Urbana, IL 61801 USA; Key Laboratory of Synthetic Biology, Institute of Plant Physiology and Ecology, Shanghai Institutes for Biological Sciences, Chinese Academy of Sciences, Shanghai, 200032 China; Molecular Biology Department, Myriant Corporation, 66 Cummings Park, Woburn, MA 01801 England; Departments of Chemistry, Biochemistry, and Bioengineering, Institute for Genomic Biology, University of Illinois at Urbana-Champaign, Urbana, IL 61801 USA

**Keywords:** Succinic acid, Acid tolerance, *Issatchenkia orientalis*, Metabolic engineering

## Abstract

**Background:**

Recent advances in synthesizing valuable chemicals such as organic acids from low-cost renewable biomass through microbial fermentation have attracted great attention. However, the toxicity of organic acids presents a key challenge to the development of an economically viable fermentation process. Therefore, a platform organism that not only produces organic acids but also tolerates the associated toxicity is highly desirable.

**Results:**

Here we report the discovery, characterization, and engineering of a yeast strain, *Issatchenkia orientalis* SD108, that is tolerant to low pH and high concentration of organic acids. This strain demonstrated a higher tolerance compared to *I. orientalis* ATCC 24210 and Classic Distiller’s Turbo yeast. In order to explore SD108 as a potential platform organism for organic acid production, we determined its draft genome sequence and use the sequencing information to guide pathway design. As proof of concept, an engineered four-gene expression cassette related to the reductive TCA cycle was assembled and integrated into the genome of a uracil auxotroph of SD108. The resulting strain was able to produce succinic acid with a titer of 11.63 g/L, yield of 0.12 g/g, and productivity of 0.11 g/L · h in batch cultures using shake flasks.

**Conclusions:**

The high tolerance of *I. orientalis* SD108 towards multiple important organic acids makes it a highly attractive organism as a platform host for producing this group of compounds as it will reduce production cost, facilitate downstream processing, and serve as a host for construction of production strains with both pH and specific anion tolerance.

**Electronic supplementary material:**

The online version of this article (doi:10.1186/s12934-014-0121-4) contains supplementary material, which is available to authorized users.

## Background

Largely owing to the concerns with sustainability, global climate change, and energy security, the use of microorganisms to convert renewable biomass to fuels and chemicals has become increasingly attractive. In 2004, the US Department of Energy (DOE) described twelve platform chemicals that could be produced from renewable biomass in a biorefinery, eight of which were organic acids [1]. The wide application of organic acids as platform chemicals, alongside the relatively few enzymatic steps required for their production, has led to intensive investigation into their microbial synthesis. One example is succinic acid, which is a precursor of many industrially important chemicals such as 1,4-butanediol, tetrahydrofuran, γ-butyrolactone and various pyrrolidinone derivatives [2]. Other organic acids of interest include itaconic acid, adipic acid and acetic acid. The major applications of itaconic acid include the use as a copolymer with acrylic acid and the conversion to many commodity and specialty chemicals including pyrrolidones and 2-methyl-1,4-butanediol [1]. Although they are not among the twelve platform chemicals, adipic acid is a key C6 dicarboxylic acid ingredient in the production of nylon 6,6 and thermoplastic polyurethanes, and has an annual market of $6.3 billion [3], whereas acetic acid is one of the common inhibitors present in lignocellulose hydrolysates, which can lead to severe growth inhibition even at low concentrations [4].

For an economically viable process, a platform organism that not only produces a high level of target organic acid but also tolerates the associated toxicity is highly desired [5]. Recently, we isolated a yeast strain, named as SD108, and identified it as *Issatchenkia orientalis* by DNA sequencing of its 26S ribosomal RNA genes. Due to its ability to grow at low pH, *I. orientalis* has been used in ethanol fermentation at pH 2 [6] and could grow on the saccharification products hydrolyzed from lignocellulosic biomass by sulfuric acid [7]. Cargill Inc. (Wayzata, MN) also used *I. orientalis* for lactic acid production in unbuffered cultures and obtained production rates and yields similar to those of traditional bacterial lactic acid processes [8]. With its potential as a platform organism to produce organic acids, further investigation of this organism is needed.

In this study, we provide a biochemical characterization of *I. orientalis* SD108 and demonstrate that this strain has an extremely high tolerance towards multiple organic acids identified by DOE as platform chemicals. In addition, we determined the draft genome sequence of SD108 via 454 *de novo* sequencing, offering a resource not only for gaining further insights into the properties of this organism, but also for designing strategies to alter its metabolism for production of chemicals. As proof of concept, four genes from the reductive TCA pathway were assembled and integrated to the genome of a uracil auxotroph strain of *I. orientalis* SD108. The resulting strain exhibited significantly improved titer, yield and productivity of succinic acid production. These results suggest SD108 is a promising host for industrial production of organic acids.

## Results

### Characterization of sugar utilization by *I. orientalis* SD108

**Figure 1:**
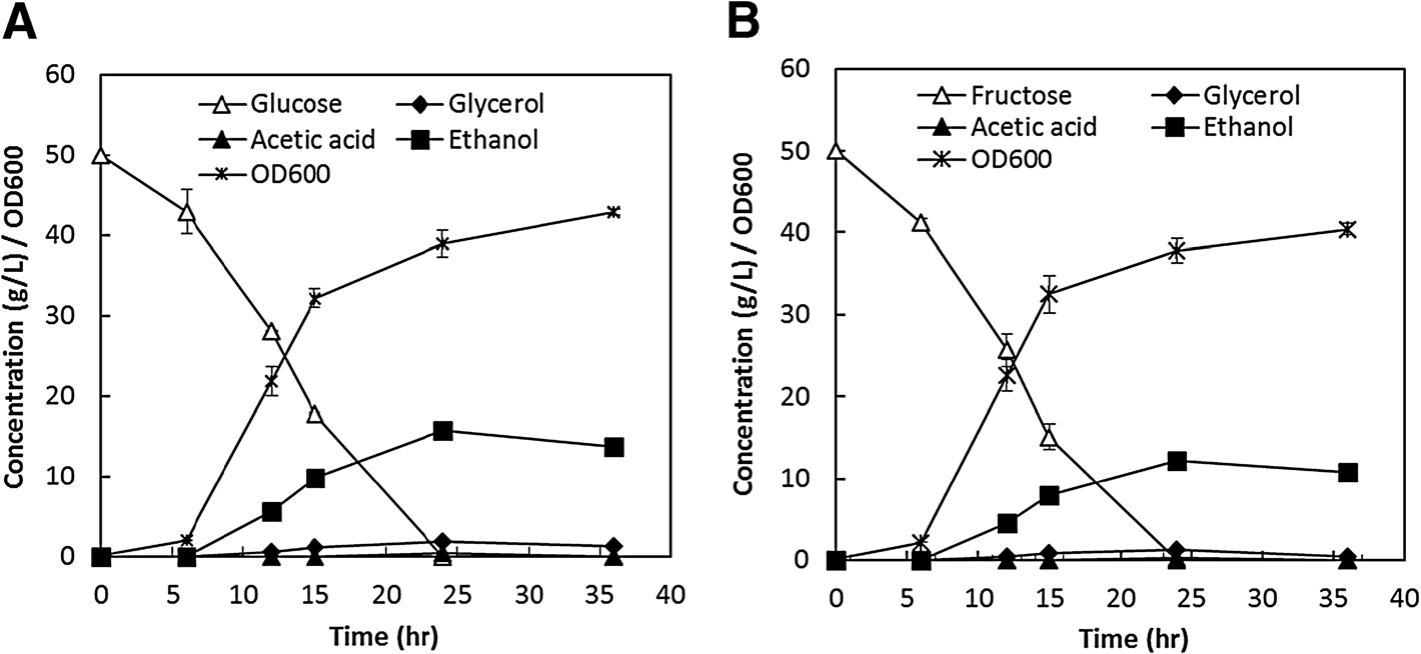
**Characterization of sugar utilization in** ***I. orientalis*** **SD108. (A)** Batch cultures using shake flasks profile of SD108 in SC medium containing 50 g/L of glucose. **(B)** Batch cultures using shake flasks profile of SD108 in SC medium containing 50 g/L of fructose.

The basic features of *I. orientalis* SD108 as a potential platform organism were first investigated. Batch cultures using shake flasks were performed at 30°C in synthetic complete (SC) medium under oxygen-limited condition. *I. orientalis* SD108 can ferment fructose as efficiently as glucose (Figure 1 and Additional file 1: Figure S1). The maximal specific growth rates of SD108 on 5 g/L of glucose and 5 g/L of fructose were 0.62 h^−1^ and 0.65 h^−1^ respectively (Additional file 1: Figure S1). Shake flask culture profiles of SD108 were further determined using 50 g/L glucose and 50 g/L fructose as carbon source, respectively. As shown in Figure 1A, SD108 was able to consume all the glucose within 24 h. The titer of glycerol was below 2 g/L. Acetic acid was not detected during most of the shake flask culture, except for 0.36 g/L at 24 h. An ethanol yield of 0.31 g/g glucose was achieved after 24 h. With fructose as the sole carbon source, the shake flask culture profile was similar to that with glucose. An ethanol yield of 0.24 g/g fructose was achieved after 24 h, which was 23% less than that of glucose (Figure 1A and B). In addition to glucose and fructose, other sugar components found in different types of biomass substrates including xylose, arabinose, cellobiose, galactose and sucrose [9–12] were tested as potential carbon sources. No consumption of these sugars was observed both at low (5 g/L) and high (50 g/L) concentrations.

### Tolerance towards organic acids

**Figure 2:**
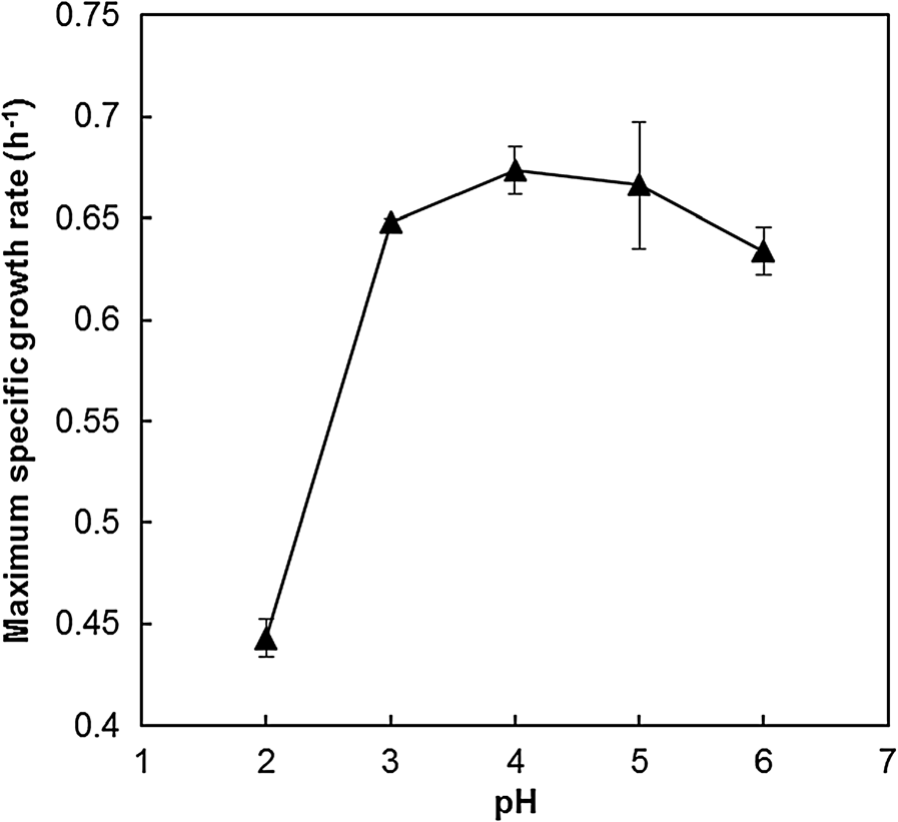
**Maximum specific growth rates of** ***I. orientalis*** **SD108 in SC medium at different starting pH values.**

The pH range and optimum pH for growth of strain SD108 were first examined from pH 0 to 6 using 50 g/L of glucose as carbon source. While it did not exhibit growth at pH 0 and 1, a wide optimum pH range of this strain was observed between pH 3 and 6, with a maximum specific growth rate of 0.67 h^−1^ (Figure 2 and Additional file 1: Figure S2). Given that the pKa values for most building block organic acids range from 3 to 5, the pH of a fermentation culture is expected to decrease to around 2.0 for acid titers of 50 g/L. The resultant acidity would possibly have little effect on cell growth of this strain.

**Figure 3:**
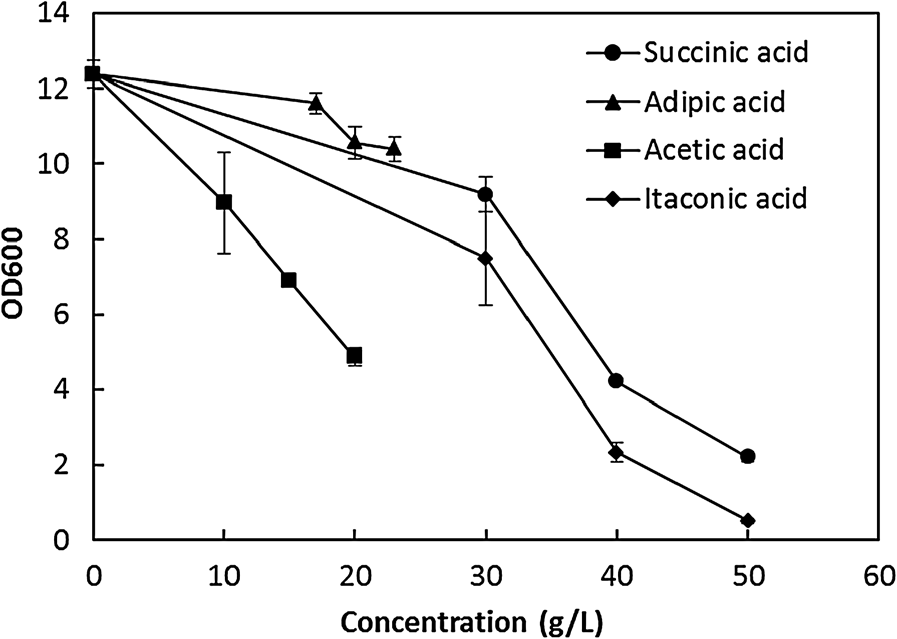
**Cell growth of** ***I. orientalis*** **SD108 after 10 h in SC medium containing various organic acids.** All the initial pH values were 5.6.

To evaluate the organic acid tolerance of SD108, four organic acids were selected: succinic acid, itaconic acid, adipic acid, and acetic acid. Cell growth of SD108 in the presence of different concentrations of these organic acids was analyzed. To eliminate initial pH discrepancies between these organic acids, all the starting pH values were adjusted to 5.6, which is in the optimal pH range of this strain (Figure 2). As shown in Figure 3, the OD_600_ values of SD108 after 10 h were greater than the initial value of 0.2 under all the tested conditions. Finally, 30 g/L of succinic acid, 30 g/L of itaconic acid, 23 g/L of adipic acid and 20 g/L of acetic acid, under which concentrations SD108 exhibited clear growth, were selected for further analysis (Figure 3).

**Figure 4:**
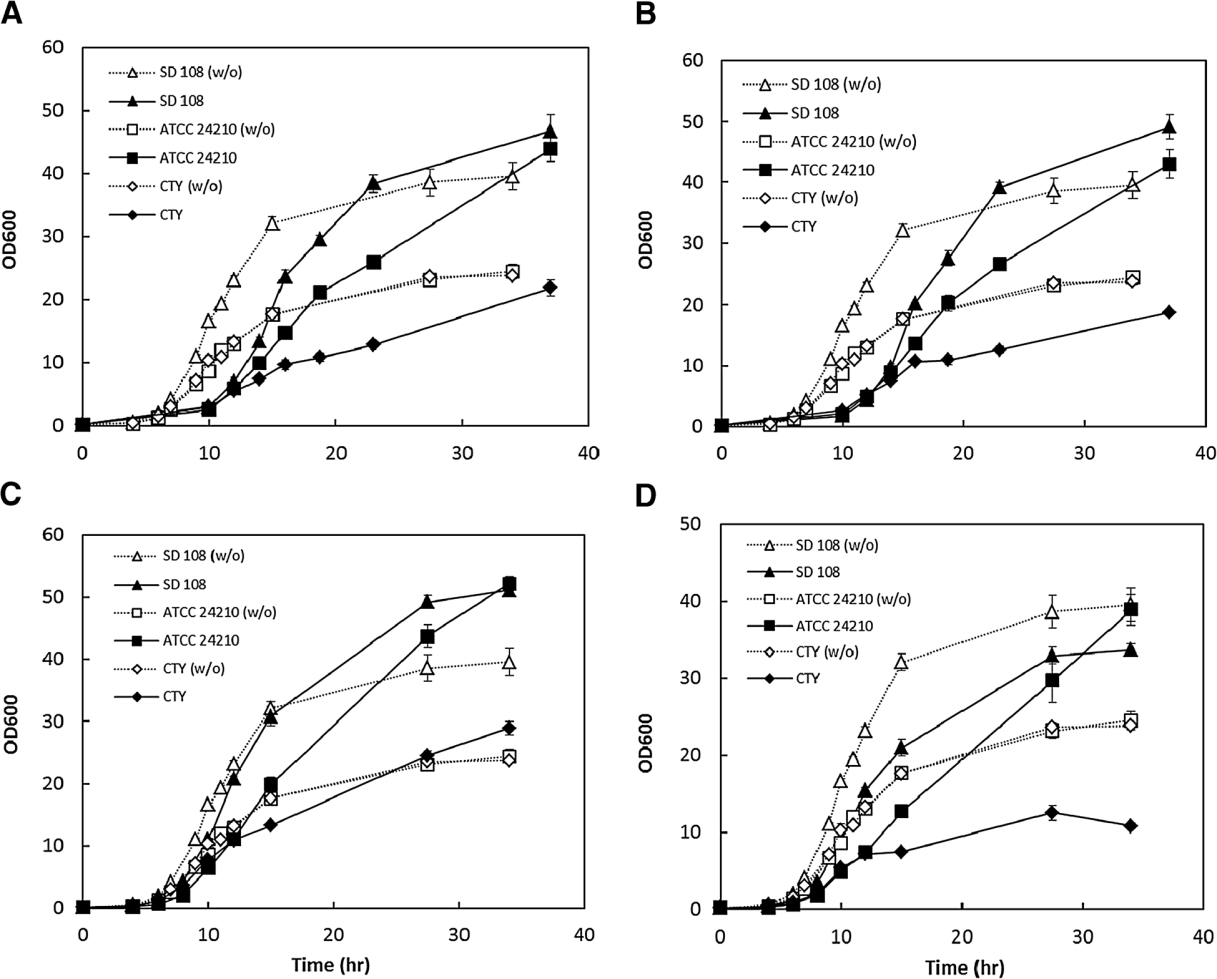
**Cell growth of the strains in SC medium containing different organic acids.** All the initial pH values were 5.6. SD 108, *I. orientalis* SD 108; ATCC24210, *I. orientalis* ATCC 24210; CTY, Classic Distiller’s Turbo Yeast; w/o, without any organic acid added. **(A)** 30 g/L of succinic acid; **(B)** 30 g/L of itaconic acid; **(C)** 23 g/L of adipic acid; **(D)** 20 g/L of acetic acid.

In order to gauge the organic acid tolerance performance of SD108, another strain of *I. orientalis*, *I. orientalis* ATCC 24210, and a fast-growing industrial yeast, Classic Distiller’s Turbo yeast, were chosen for comparison. Classic Distiller’s Turbo yeast exhibits higher tolerance towards multiple alcohols and inhibitors contained in lignocellulose hydrolysates (e.g., ethanol, n-butanol and furfural) than a series of *Saccharomyces cerevisiae* laboratory strains including *S. cerevisiae* W303a, *S. cerevisiae* BY4741 and *S. cerevisiae* CEN.PK 113-7D (data not shown). No significant difference in the lag phase was found among these three strains in the presence of various organic acids, although the lag phase of the strains in the presence of 30 g/L of succinic acid or 30 g/L of itaconic acid was 6 hours longer than those observed in 23 g/L of adipic acid or 20 g/L of acetic acid (Figure 4). As shown in Table 1, the maximum specific growth rates of both *I. orientalis* ATCC 24210 and SD108 were 0.65 h^−1^ in the cultures without any supplemented organic acid, which are 12% higher than Classic Distiller’s Turbo yeast. SD108 exhibited maximum specific growth rates of 0.37, 0.43, 0.58 and 0.55 h^−1^ in the cultures with 30 g/L of succinic acid, 30 g/L of itaconic acid, 23 g/L of adipic acid and 20 g/L of acetic acid, respectively. These rates were 68%, 65%, 12% and 25% higher than Classic Distiller’s Turbo yeast under such conditions. It was noted that strain SD108 exhibits significantly improved tolerance over strain *I. orientalis* ATCC 24210 in the presence of 30 g/L itaconic acid, 23 g/L adipic acid and 20 g/L acetic acid, where the maximum specific growth rates of SD108 were 16%, 5% and 10% higher than those of *I. orientalis* ATCC 24210, respectively (Table 1).

**Table 1:** **Maximum specific growth rate of strains in SC medium containing 20 g**/**L of acetic acid**, **23 g**/**L of adipic acid**, **30 g**/**L of itaconic acid and 30 g**/**L of succinic acid**, **respectively**

**Strains**	**Maximum specific growth rate** **(h** ^**−1**^ **)**				
	**None**	**Succinic acid**	**Itaconic acid**	**Adipic acid**	**Acetic acid**
Classic Distiller’s Turbo Yeast	0.58 ± 0.02	0.22 ± 0.01	0.26 ± 0.01	0.52 ± 0.01	0.44 ± 0.01
*I. orientalis* ATCC 24210	0.65 ± 0.02	0.34 ± 0.02	0.37 ± 0.03	0.55 ± 0.01	0.50 ± 0.03
*I. orientalis* SD108	0.65 ± 0.01^a^	0.37 ± 0.01^a^	0.43 ± 0.00^a,b^	0.58 ± 0.01^a,b^	0.55 ± 0.01^a,b^

All the initial pH values were 5.6.

^a^The maximum specific growth rate of strain *I. orientalis* SD108 was statistically significant over that of strain Classic Distiller’s Turbo Yeast (*P* < 0.05) as determined by the Student t-test; ^b^The maximum specific growth rate of strain *I. orientalis* SD108 was statistically significant over that of strain *I. orientalis* ATCC 24210 (*P* < 0.05) as determined by the Student t-test.

### Genome sequencing and ploidy

The above features emphasize the biotechnological potential of *I. orientalis* and reinforce a need for developing genetic manipulation tools for this strain. The availability of genome sequence of SD108 becomes important, which may serve as the molecular basis for designing genetic and metabolic engineering strategies. A draft genome sequence was established by whole-genome-shotgun and paired-end sequencing using the Genome Sequencer FLX + system and subsequent data assembly using the GS De Novo Assembler version 2.6 from 454 Life Sciences. There are 5,093 predicted genes, more than 85% of which have homologs in *Pichia stipitis* and *S. cerevisiae*. A schematic of predicted genes in *I. orientalis* SD108, related to carbohydrates utilization, glycolysis, pentose phosphate pathway, pyruvate metabolism, TCA cycle and glyoxylate shunt pathway, is shown in Additional file 1: Figure S3. In addition, relying on chromosomal DNA staining and flow cytometry [13], SD108 was determined as a diploid strain. More details about genome sequencing and ploidy determination are shown in supplementary materials.

### Generation of a uracil auxotroph *I. orientalis* SD108 strain

We first sought to generate a uracil auxotroph strain as a recipient strain for future genetic manipulation with uracil auxotrophy as selection marker. The regions flanking the *ura3* open reading frame were separated amplified and spliced via overlap extension PCR as a deletion fragment. After transforming 5 μg of this deletion fragment into SD108, transformants were spread on SC + FOA plates. After incubation at 30°C for at least one week, twenty colonies appeared on the SC + FOA plate and each colony was restreaked on a SC-URA plate and a new SC + FOA plate in parallel. Eight of the twenty candidates could only grow on the SC + FOA plate (Additional file 1: Figure S5A) and PCR analysis of their isolated genomic DNAs confirmed the loss of both copies of *ura3*. Transforming these strains with a wild type *ura3* gene, yielding strain IoΔura3 + ura3, could complement the loss of *ura3* function (Additional file 1: Figure S5B). As expected, only one locus was repaired, which was confirmed by PCR analysis (Additional file 1: Figure S5C).

### Metabolic engineering of succinic acid producing *I. orientalis* SD108 strain

**Figure 5:**
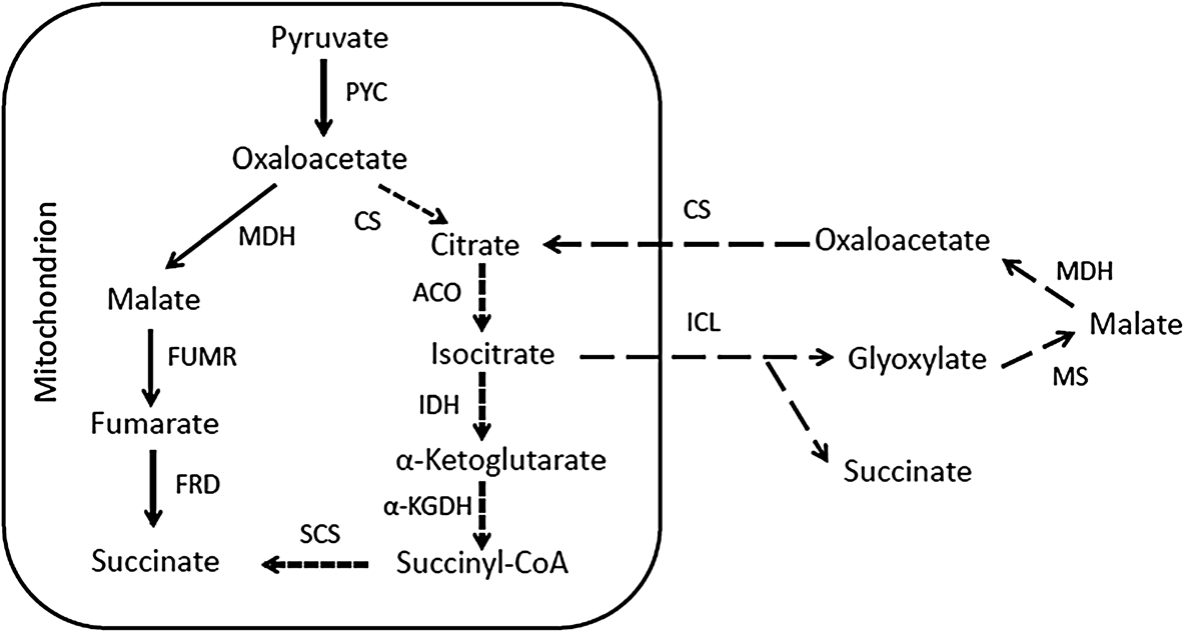
**Three fermentation pathways for succinate production.** The reductive TCA, oxidative TCA and glyoxylate shunt pathway were shown by solid, short dashed and long dashed arrows respectively. PYC, pyruvate carboxylase; MDH, malate dehydrogenase; FUMR, fumarase; FRD, fumarate reductase; CS, citrate synthase; ACO, aconitase; IDH, isocitrate dehydrogenase; α-KGDH, α-ketoglutarate; SCS, succinyl coenzyme A synthetase; ICL, isocitrate lyase; MS, malate synthase.

As the next step to test whether SD108 could be a promising host for producing organic acids, we chose to work on the biosynthesis of succinic acid. The reductive TCA cycle, oxidative TCA cycle and glyoxylate shunt pathway are the three primary fermentation pathways for producing succinic acid (Figure 5), among which the reductive TCA cycle gives the highest theoretical yield on glucose (1.31 g/g) [14]. Based on the reductive TCA cycle, genes *pyc*, *mdh*, *fumr* and *frd*, encoding pyruvate carboxylase, malate dehydrogenase, fumarase and fumarate reductase, respectively, are the most important targets for applying metabolic engineering strategies to improve succinic acid production [14,15]. In addition, to avoid the potential issue with succinic acid being exported across the inner membranes (e.g., from mitochondria to cytoplasm), enzymes that are likely expressed in the cytoplasm became our first choice. According to the genome annotation, gene *JL09*_*g1614* and *JL09*_*g1983* are predicted to be the *pyc* and *fumr* genes, respectively. Three types of malate dehydrogenase – mitochondrial malate dehydrogenase, cytoplasmic malate dehydrogenase, and peroxisomal malate dehydrogenase – are found in yeast, which are named as MDH1, MDH2 and MDH3, respectively [16]. Genes *JL09*_*g238*, *JL09*_*g1975* and *JL09*_*g4199* are predicted as *mdh*. According to the amino acid identities with MDHs in *S. cerevisiae* S288c, all of the predicted MDHs share amino acid identities (0.37 ~ 0.42) similar to MDH2 in *S. cerevisiae* S288c. Therefore, gene *JL09*_*g4199* was arbitrarily chosen as *mdh*. In addition, since none of the genes was identified as *frd* in the sequenced genome, we decided to test the previously reported codon-optimized FRD in *S. cerevisiae* [15] (Table 2). To ensure that all these enzymes would be co-expressed in the cytoplasm, potential signal peptides were removed (C-terminal PKL of MDH, N-terminal 15 amino acids of FUMR, and C-terminal SKI of FRD) according to the sequence information [15].

**Table 2:** **Strains and plasmids used in this study**

**Strains or plasmids**	**Characteristics**	**Reference or source**
*Strains*		
*I. orientalis*		
ATCC 24210	Wild type	NRRL
SD108	Wild type	This study
IoΔura3	SD108/*ura3Δ*	This study
IoΔura3 + ura3	IoΔura3/*ura3Δ*::ura3	This study
IoΔura3 + SA	IoΔura3/*ura3Δ*::ura3-succinic acid biosynthetic pathway	This study
*S. cerevisiae*		
BY4741	*MATa his3Δ0 leu2Δ0 met15Δ0 ura3Δ0*	Reference [17]
PRT238	Wild type	Offered by Dr. Peter Orlean
Classic Distiller’s Turbo Yeast	Wild type	Reference [18]
*E. coli*		
DH5α	General cloning host	Takara
BW25141	Cloning host	Provided by Professor William Metcalf
*Plasmids*		
pPK2	ColE1 ORI, Amp^r^, *C. parapsilosis* ARS7 ORI, *CpURA3*	Reference [19]
pPICZαA	With gene *ble*, encoding the zeocin resistance gene, under the control of *Saccharomyces cerevisiae* TEF1 promoter.	Life Technologies
pPK2-zeocin	Derived from pPK2, with zeocin expression cassette inserted at the region of ura3 expression cassette	This study
pXZ1	Derived from pPK2, with *C. parapsilosis* ARS7 ORI deleted.	This study
pXZ2	Plasmid which can be maintained in SD108	This study
pFRD	With codon optimized *frd* gene for *S. cerevisiae* (Genbank accession number AAN40014)	Synthesized by DNA 2.0
pRS415	Yeast centromere with LEU2 marker	NEB
pRS415-A	Derived from pRS415, with *ura3* expression cassette added	This study
pRS415-B	Derived from pRS415, with *pyc* expression cassette added	This study
pRS415-C	Derived from pRS415, with *mdh* and *fumr* expression cassette added	This study
pRS415-D	Derived from pRS415, with *frd* expression cassette added	This study
pRS415-E	Derived from pRS415-A, with *pyc*, *mdh*, *fumr* and *frd* expression cassettes added	This study

**Figure 6:**
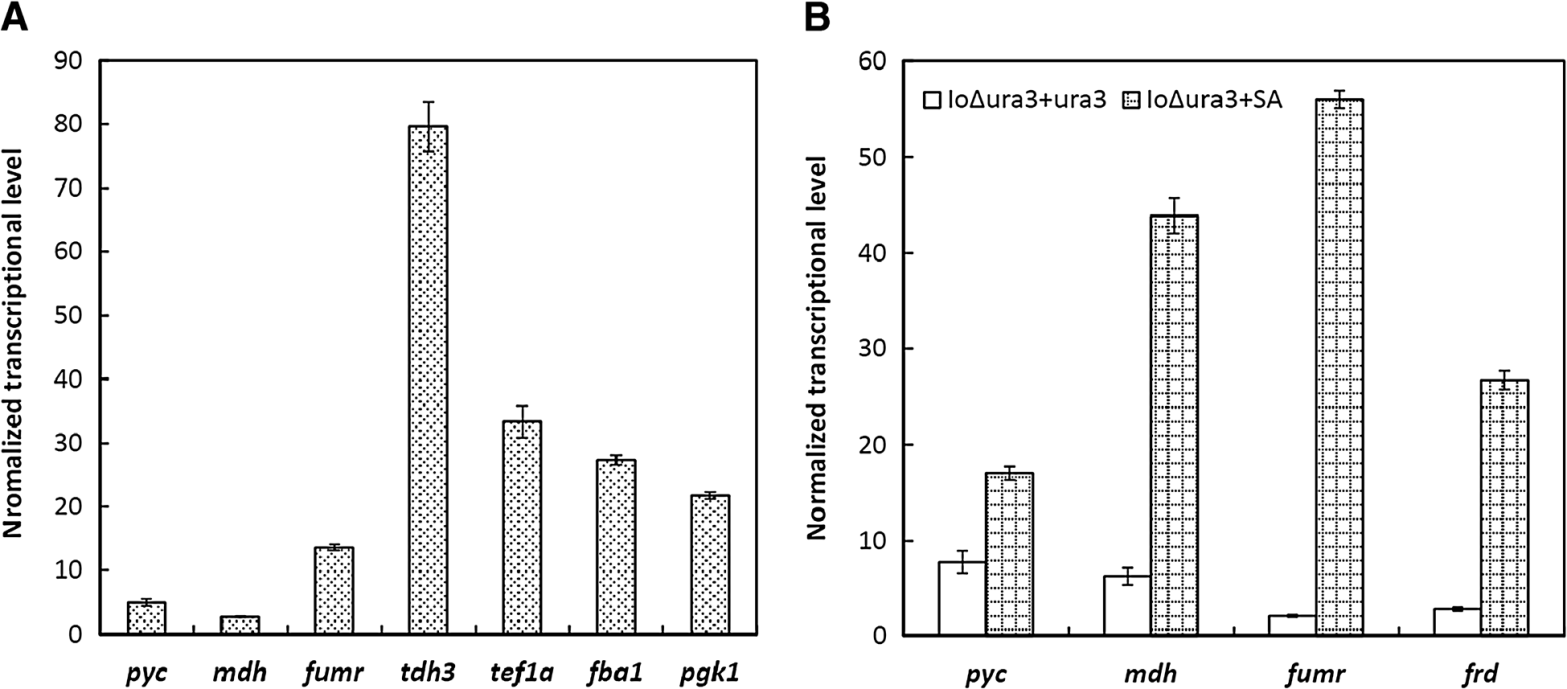
**Transcritional profiles of the succinic acid pathway**, **gene** ***alg9*** **was used as the internal control. (A)** Transcriptional levels of the succinic acid pathway at 24 h in strain IoΔura3 + ura3. Shake flask culture experiments were carried out in the SC-URA medium containing 50 g/L glucose; **(B)** Transcriptional levels of genes *pyc*, *mdh*, *fumr* and *frd* at 48 h in the IoΔura3 + ura3 and IoΔura3 + SA strains. Shake flask culture experiments were carried out in the SC-URA medium containing 50 g/L glucose and 25.52 g/L calcium carbonate.

In order to obtain high transcription levels, the *pyc*, *mdh*, *fumr* and *frd* genes were individually cloned to downstream of strong promoters *fba1p*, *tef1ap*, *pgk1p* and *tdh3p*, respectively. These promoters were chosen due to the relatively high transcription levels of the corresponding native genes under our cultivation conditions determined via real-time PCR analysis (Figure 6A). When SC-URA medium containing 50 g/L glucose and 25.52 g/L calcium carbonate was used as the shake flask culture broth, the final engineered strain IoΔura3 + SA consumed 49.72 ± 0.01 g/L glucose and produced 2.61 ± 0.29 g/L of succinic acid within 24 h, while strain IoΔura3 + ura3 consumed 49.71 ± 0.01 g/L glucose and only produced 0.37 ± 0.01 g/L of succinic acid under the same condition. The enhancement of the transcriptional levels of *pyc*, *mdh*, *fumr* and *frd* was further confirmed in strain IoΔura3 + SA (Figure 6B).

**Figure 7:**
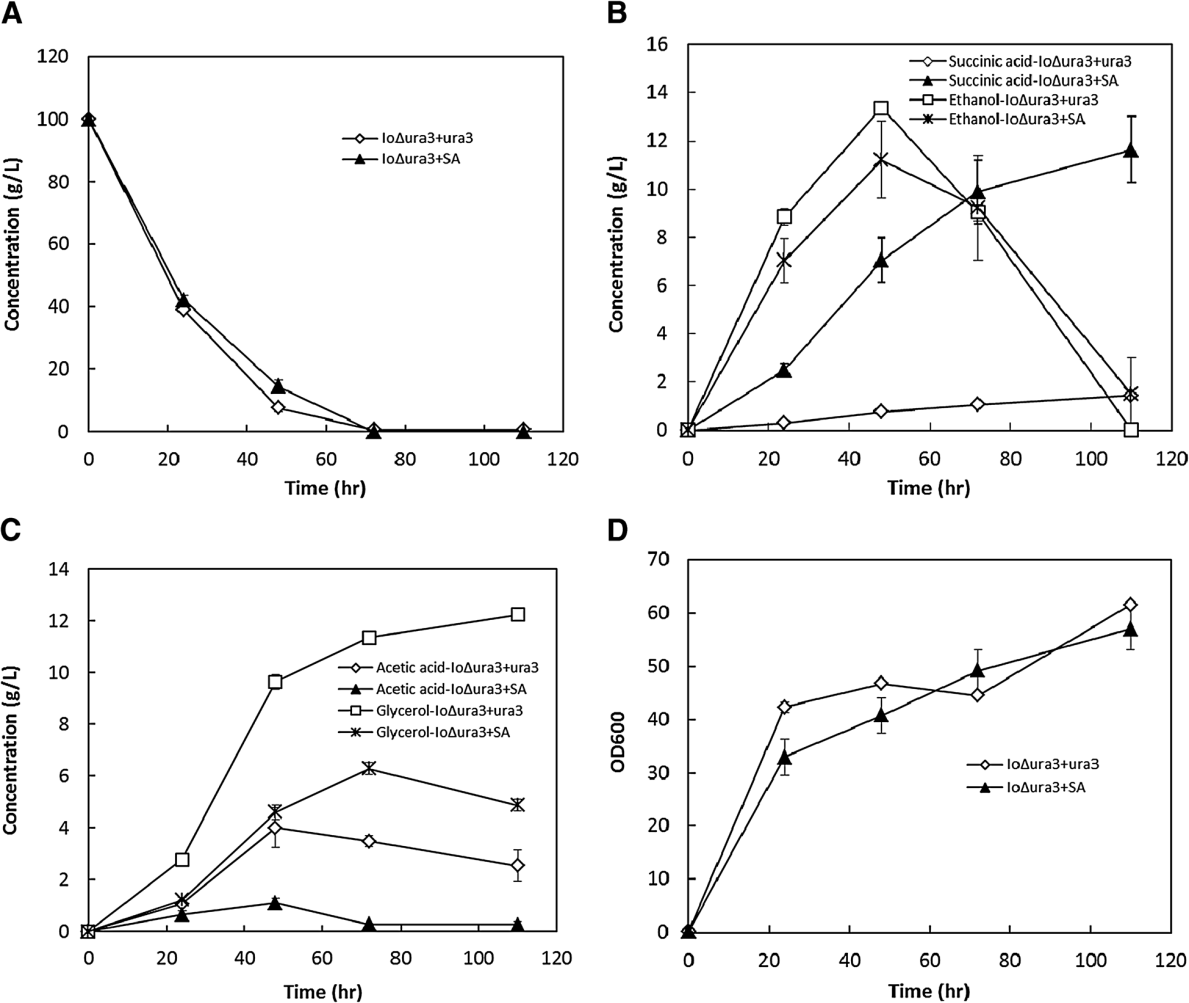
**Growth and metabolite profiles in batch culture using shake flasks of strain IoΔura3** **+ ura3 and strain IoΔura3** **+ SA in the SC-** **URA medium containing 100 g/** **L glucose. (A)** Sugar consumption; **(B)** Succinic acid and ethanol concentration; **(C)** Acetic acid and glycerol concentration; **(D)** Cell growth.

To further compare the shake flask culture characteristics, IoΔura3 + SA strains were tested in SC-URA medium containing 100 g/L glucose and 25.52 g/L calcium carbonate. No significant differences were found in glucose consumption, growth and ethanol production between strain IoΔura3 + SA and strain IoΔura3 + ura3 over the entire shake flask culture course (Figure 7A, B and D). Strain IoΔura3 + SA was able to consume all glucose and produce 11.63 ± 1.38 g/L succinic acid at 110 h, whereas strain IoΔura3 + ura3 consumed 99.29 ± 0.08 g/L glucose and yielded 1.43 ± 0.04 g/L succinic acid during the same period (Figure 7B). Because of the neutralization by calcium carbonate, the final pH of strain IoΔura3 + SA was only 1.78 units lower than that of strain IoΔura3 + ura3 (5.08 ± 0.12 *vs*. 6.86 ± 0.74). Notably, the glycerol produced by strain IoΔura3 + SA was much lower than strain IoΔura3 + ura3 (Figure 7C), which may be attributed to the amount of NADH needed by the reductive TCA cycle [14,15,20]. In addition, the acetic acid produced by strain IoΔura3 + SA was also lower than that of strain IoΔura3 + ura3 (Figure 7C). Although formation of acetic acid may be due to the regeneration of reducing equivalents, the substrate oxaloacetate required by the reductive TCA cycle may become a driving force for directing pyruvate into oxaloacetate instead of acetic acid [21]. As a result, the succinic acid titer, yield on glucose, and productivity of strain IoΔura3 + SA (11.63 g/L, 0.12 g/g and 0.11 g/L · h) were much higher than those of strain IoΔura3 + ura3 (1.43 g/L, 0.01 g/g and 0.01 g/L · h).

## Discussion

Product toxicity is a lingering problem in developing economical fermentative processes for production of organic acids despite decades of research. In addition, economically viable biorefinery operations require the use of cheap substrates such as lignocellulosic hydrolysates. This necessitates the use of strains with two properties: first, the ability to utilize various biomass sugars, and second, the ability to tolerate inhibitors contained in lignocellulosic hydrolysates. Although SD108 cannot intrinsically utilize many sugars released from lignocellulosic hydrolysates, it should be feasible to introduce the sugar utilization pathways from related species (e.g. *Pichia kudriavzevii* M12 [22]) into this strain, similar to strategies which have been successfully applied in *S. cerevisiae* [23–25]. Moreover, various by-products, mainly organic acids, are inevitably generated during hydrolysis and exert negative effects on the host [26]. In these scenarios, platform organisms which tolerate multiple organic acids have an obvious advantage as they can utilize hydrolysates more robustly. The high tolerance of *I. orientalis* SD108 towards multiple important organic acids makes it a highly attractive platform host for producing this group of compounds. In addition, the doubling time of SD108 was 11% shorter than the fast growing yeast, Classic Distiller’s Turbo Yeast (1.06 h *vs*. 1.19 h) (Table 1 and Figure 4). This means that more cells of SD108 are available during the same time period, and thus the high productivity desired in industrial processes may be achieved more easily.

For such a potential platform organism, the availability of its genome sequence is essential. It will offer further insights into its properties, and more importantly, facilitate the genetic and metabolic engineering studies. The molecular mechanisms underlying the ability of *I. orientalis* to resist the lethal effects of a spectrum of organic acids have not been identified. Normally, acid stress will induce complex transcriptional responses and intricate processes that relate to a set of genes. Hence genome-wide analysis of the transcriptome and proteome are usually needed to identify the genes involved in stress response [27,28]. Known tolerance mechanisms used by *S. cerevisiae* and *E. coli* to cope with acidic environments include, but are not limited to, inducing expression of stress genes, changing membrane composition, transporting protons out of the cell, and consuming protons via decarboxylation reactions [27,29–32]. By comparison of previously identified determinants of acid tolerance, candidate genes related to acid tolerance in *I. orientalis* SD108 are summarized in Additional file 1: Table S5. Our draft genome sequence offers a basis for designing further functional genomics studies to investigate the mechanisms underlying acid tolerance.

Succinic acid production is a specific example of exploiting genome information to rewire metabolic pathways. By integration of endogenous genes *pyc*, *mdh*, *fumr* and an exogenous gene *frd* driven by endogenous strong promoters, the resultant strain exhibited significant improvement of the titer, yield and productivity (Figure 7). The reductive TCA cycle is the most interesting pathway for succinic acid production. While it provides the highest succinic acid yield on glucose as compared with the other two pathways, the net deficit in reducing power (NADH) poses a challenge for metabolic engineering [14]. Strategies to address the redox imbalance include either combining the oxidative TCA and/or glyoxylate shunt pathways with the reductive TCA cycle, supplying NADH and direct carbon flux into succinic acid, or introducing other pathways for NADH supply (e.g., glucose-6-phosphate dehydrogenase) [33,34]. In addition, blocking byproduct production (e.g., glycerol, ethanol and acetate) and deletion of the reverse reactions of the reductive TCA cycle (e.g., from succinate to fumarate, which is catalyzed by succinate dehydrogenase) should also be considered in future plans.

In addition to chromosomal integration, an episomal plasmid based expression system is desirable in a potential platform organism. Neither 2 μ nor CEN ARS sequences, which are commonly used as the origin of replication in *S. cerevisiae*, could be maintained in SD108. We were able to successfully identify a functional ARS from the digested genomic DNA of SD108 (Additional file 1: Figure S6). The plasmid containing this 91 bp sequence could express zeocin resistance marker and can be maintained throughout the cultivation process, providing another viable option to express the target pathway in this host. We are currently determining the copy number of such an ARS and testing the succinic acid production if the associated genes are expressed on an episomal plasmid. In addition, SD108 is diploid, which makes gene deletion inconvenient. The plasmid-based expression in SD108 should facilitate the deployment of recently developed powerful genome editing tools (e.g., TALEN and CRISPR) [35–39], which may delete multiple copies of one gene or different genes simultaneously.

## Conclusions

The organic acid biorefinery industry demands a better “microbial factory” that exhibits an excellent tolerance to moderately low pH and high product titer conditions, and can grow and produce the target organic acid at high rates. *I. orientalis* SD108 indeed demonstrates an exceptional tolerance towards multiple industrially important organic acids. Our biochemical and genetic characterization provides a resource to identify novel candidate genes coding for proteins involved in acid tolerance and offers a basis to design future strategies to evaluate its metabolic capabilities and biotechnologically important features. Based on the genome information, genetic tools for SD108 have been developed and metabolic engineering strategies have successfully been applied for increasing succinic acid production, which further makes this strain more attractive.

## Materials and methods

### Strains and growth media

The strains and plasmids used in this study are listed in Table 2. Cells were grown in liquid SC [18], SC-URA or SC-LEU medium supplemented with various sugars as the carbon source or on solid YPAD medium (1% yeast extract, 2% peptone, 0.01% adenine hemisulfate, 2% glucose and 2% agar) unless otherwise noted. The initial pH value of SC culture was adjusted to 5.6 using 12 M NaOH, and was not controlled during shake flask culture. For isolation and identification of SD108, see Additional file 1: Supplementary data for details.

### Tolerance assay

For calculating maximum specific growth rates of *I. orientalis* SD108 at different starting pH values, stationary-phase cells grown in SC medium [18] were transferred into 5 mL of SC medium containing 50 g/L glucose in a 15 mL round-bottom Falcon tube (30°C, 250 rpm). The initial pH values were adjusted to 0, 1, 2, 3, 4, 5 or 6 by using 12 M HCl or 12 M NaOH, citric acid-sodium citrate buffer solutions were used to maintain the pH (http://www.sigmaaldrich.com/life-science/core-bioreagents/biological-buffers/learning-center/buffer-reference-center.html) (Additional file 1: Figure S2). To determine cell growth of *I. orientalis* SD108 in SC medium containing various organic acids, stationary-phase cells grown in SC medium were transferred into 5 mL of SC medium containing 50 g/L glucose and various concentrations of organic acids in a 15 mL round-bottom Falcon tube (30°C, 250 rpm). Cell densities after 10 hours of growth were measured. For the organic acid tolerance assay, stationary-phase cells grown in SC medium containing 50 g/L glucose were transferred into 20 mL of fresh SC medium containing 50 g/L glucose and various organic acids at appropriate concentrations in 125 mL non-baffled shake flasks. Cells were grown under oxygen-limited conditions as described previously (30°C, 100 rpm) [18]. The initial OD_600_ for various tolerance assays were 0.2. The initial pH values were adjusted to 5.6 using 12 M NaOH except as described elsewhere.

### DNA transformation of SD108 and its derived strains

DNA transformation of SD108 and its derived strains was carried out using the method developed by Gietz and Schiestl [40]. See Additional file 1: Supplementary data for details.

### Generation of a uracil auxotroph SD108 strain

Genomic DNA from *I. orientalis* SD108 was isolated with the Wizard Genomic DNA Purification Kit (Promega, Madison, WI). An upstream homology arm of 543 bp and a downstream homology arm of 279 bp were PCR-amplified separately from the genomic DNA using primers Ura-p-up/Ura-p-dn and Ura-t-up/Ura-t-dn, respectively (Additional file 1: Table S1). These arms were spliced into a *ura3* deletion fragment via overlap extension PCR [41]. A total of 5 μg of the *ura3* deletion fragment was used to delete both copies of *ura3* simultaneously. DNA transformation was carried out with the time for heat shock being 90 min. Transformants were spread on SC + FOA plates and incubated at 30°C for approximately 10 days. Transformation protocol was further optimized using the uracil auxotroph SD108 strain as a starting strain. See Additional file 1: Supplementary data for details.

### Assembly of the reductive TCA pathway for succinic acid production

Pathway construction was carried out using the DNA assembler method [42]. See Additional file 1: Supplementary data for details.

### RNA preparation and quantitative PCR analysis

Samples for qPCR were collected from 20 mL of SC-URA medium containing 50 g/L glucose or 50 g/L glucose plus 25.52 g/L calcium carbonate. RNA preparation, generation of cDNA and real-time PCR analysis were performed as described previously [43]. The *alg9* gene was used as an internal control, commonly used as the internal control for transcriptional analysis in *S. cerevisiae* [44]. The qPCR primers are listed in Additional file 1: Table S1.

### Shake flask cultures

Batch cultures using shake flasks were carried out as follows: a single colony grown on SC-URA plate was inoculated into 3 mL of SC-URA medium containing 2% glucose in a 15 mL round-bottom Falcon tube and grown until saturation (30°C, 250 rpm). About 100 μL of the stationary-phase cells were transferred into 20 mL of fresh SC-URA media containing 25.52 g/L calcium carbonate and different concentrations of glucose in 125 mL non-baffled shake flasks. Cells were grown under oxygen-limited conditions (30°C, 100 rpm). The initial OD_600_ was 0.2.

### HPLC analysis

The samples were centrifuged and the supernatants were diluted 0 to 10 times before HPLC analysis. An HPLC system equipped with a refractive index detector (Shimadzu Scientific Instruments, Columbia, MD) was used to analyze the concentrations of glucose, xylose, arabinose, cellobiose, galactose, sucrose, fructose, ethanol, acetic acid, glycerol and succinic acid in the broth. To separate all the metabolites mentioned above, an HPX-87H column (BioRad, Hercules, CA) was used as described previously [18]. The HPLC chromatogram was analyzed using the LCsolution software (Shimadzu Scientific Instruments, Columbia, MD).

## Additional file

Additional file 1:
**Supplementary Data.**

## Abbreviations

DOE: Department of energy
FOA: 5-Fluoroorotic acid
FRD: Fumarate reductase
FUMR: Fumarase
MDH: Malate dehydrogenase
PYC: Pyruvate carboxylase
SC: Synthetic complete

## References

[1] Werpy T, Petersen G: **Top Value Added Chemicals from Biomass, vol. I: Results of Screening for Potential Candidates from Sugars and Synthesis Gas.** In 2004 [http://www1.eere.energy.gov/bioenergy/pdfs/35523.pdf]

[2] Song H, Lee SY (2006). Production of succinic acid by bacterial fermentation. Enzyme Microb Technol.

[3] Beardslee T, Picataggio S: **Biological Methods for Preparing Adipic Acid.** US; 2012. patent 8241879 B2.

[4] Tanaka K, Ishii Y, Ogawa J, Shima J (2012). Enhancement of acetic acid tolerance in *Saccharomyces cerevisiae* by overexpression of the HAA1 Gene, encoding a transcriptional activator. Appl Environ Microbiol.

[5] Abbott DA, Zelle RM, Pronk JT, Van Maris AJ (2009). Metabolic engineering of *Saccharomyces cerevisiae* for production of carboxylic acids: current status and challenges. FEMS Yeast Res.

[6] Hisamatsu M, Furubayashi T, Karita S, Mishima T, Isono N (2006). Isolation and identification of a novel yeast fermenting ethanol under acidic conditions. J Appl Glycosci.

[7] Thalagala TATP, Kodama S, Mishima T, Isono N, Furujyo A, Kawasaki Y, Hisamatsu M (2009). Study on ethanol fermentation using D-glucose rich fractions obtained from lignocelluloses by a two-step extraction with sulfuric acid and *Issatchenkia orientalis* MF 121. J Appl Glycosci.

[8] Suominen P, Aristidou A, Penttila M, Ilmen M, Ruohonen L, Koivuranta K, Roberg-Perez K: **Genetically Modified Yeast of the Species*****Issatchenkia orientalis*****and Closely Relates Species, and Fermentation Processes using Same**. In 2012. patent 8,097,448.

[9] Balat M, Balat H, Oz C (2008). Progress in bioethanol processing. Prog Energy Combust Sci.

[10] Fitzpatrick M, Champagne P, Cunningham MF, Whitney RA (2010). A biorefinery processing perspective: treatment of lignocellulosic materials for the production of value-added products. Bioresour Technol.

[11] Kumar R, Singh S, Singh OV (2008). Bioconversion of lignocellulosic biomass: biochemical and molecular perspectives. J Ind Microbiol Biotechnol.

[12] Van Maris AJA, Abbott DA, Bellissimi E, van den Brink J, Kuyper M, Luttik MAH, Wisselink HW, Scheffers WA, Van Dijken JP, Pronk JT (2006). Alcoholic fermentation of carbon sources in biomass hydrolysates by *Saccharomyces cerevisiae*: current status. Anton Leeuw Int J G.

[13] Almeida AJ, Matute DR, Carmona JA, Martins M, Torres I, McEwen JG, Restrepo A, Leao C, Ludovico P, Rodrigues F (2007). Genome size and ploidy of *Paracoccidioides brasiliensis* reveals a haploid DNA content: flow cytometry and GP43 sequence analysis. Fungal Genet Biol.

[14] Finley KR, Huryta JM, Mastel BM, Mcmullin TW, Poynter GM, Rush BJ, Watts KT, Fosmer AM, Mcintosh JRVL, Brady KM: **Compositions and Methods for Succinate Production**. In 2012. patent WO2012103261 A2.

[15] Verwaal R, Wu L, Damveld AR, Sagt JMC: **Succinic Acid Production in a Eukaryotic Cell.** US; 2012. patent 20120165569 A1.

[16] Gibson N, Mcalister-Henn L (2003). Physical and genetic interactions of cytosolic malate dehydrogenase with other gluconeogenic enzymes. J Biol Chem.

[17] Radonjic M, Andrau JC, Lijnzaad P, Kemmeren P, Kockelkorn TT, Van Leenen D, Van Berkum NL, Holstege FC (2005). Genome-wide analyses reveal RNA polymerase II located upstream of genes poised for rapid response upon *S. cerevisiae* stationary phase exit. Mol Cell.

[18] Du J, Yuan Y, Si T, Lian J, Zhao H (2012). Customized optimization of metabolic pathways by combinatorial transcriptional engineering. Nucleic Acids Res.

[19] Kosa P, Gavenclakova B, Nosek J (2007). Development of a set of plasmid vectors for genetic manipulations of the pathogenic yeast *Candida parapsilosis*. Gene.

[20] Geertman JMA, Van Dijken JP, Pronk JT (2006). Engineering NADH metabolism in *Saccharomyces cerevisiae*: formate as an electron donor for glycerol production by anaerobic, glucose-limited chemostat cultures. FEMS Yeast Res.

[21] Thakker C, Zhu J, San KY, Bennett G (2011). Heterologous pyc gene expression under various natural and engineered promoters in Escherichia coli for improved succinate production. J Biotechnol.

[22] Chan GF, Gan HM, Ling HL, Rashid NA (2012). Genome sequence of *Pichia kudriavzevii* M12, a potential producer of bioethanol and phytase. Eukaryot Cell.

[23] Becker J, Boles E (2003). A modified *Saccharomyces cerevisiae* strain that consumes L-arabinose and produces ethanol. Appl Environ Microbiol.

[24] Hahn-Hagerdal B, Wahlbom CF, Gardonyi M, Van Zyl WH, Cordero Otero RR, Jonsson LJ (2001). Metabolic engineering of *Saccharomyces cerevisiae* for xylose utilization. Adv Biochem Eng Biotechnol.

[25] Kotter P, Ciriacy M (1993). Xylose fermentation by *Saccharomyces*-*cerevisiae*. Appl Microbiol Biotechnol.

[26] Palmqvist E, Hahn-Hagerdal B (2000). Fermentation of lignocellulosic hydrolysates. II: inhibitors and mechanisms of inhibition. Bioresour Technol.

[27] King T, Lucchini S, Hinton JC, Gobius K (2010). Transcriptomic analysis of *Escherichia coli* O157:H7 and K-12 cultures exposed to inorganic and organic acids in stationary phase reveals acidulant- and strain-specific acid tolerance responses. Appl Environ Microbiol.

[28] Guerreiro JF, Mira NP, Sa-Correia I (2012). Adaptive response to acetic acid in the highly resistant yeast species *Zygosaccharomyces bailii* revealed by quantitative proteomics. Proteomics.

[29] Kawahata M, Masaki K, Fujii T, Iefuji H (2006). Yeast genes involved in response to lactic acid and acetic acid: acidic conditions caused by the organic acids in *Saccharomyces cerevisiae* cultures induce expression of intracellular metal metabolism genes regulated by Aft1p. FEMS Yeast Res.

[30] Lawrence CL, Botting CH, Antrobus R, Coote PJ (2004). Evidence of a new role for the high-osmolarity glycerol mitogen-activated protein kinase pathway in yeast: regulating adaptation to citric acid stress. Mol Cell Biol.

[31] Martinez-Munoz GA, Kane P (2008). Vacuolar and plasma membrane proton pumps collaborate to achieve cytosolic pH homeostasis in yeast. J Biol Chem.

[32] Warnecke T, Gill RT (2005). Organic acid toxicity, tolerance, and production in *Escherichia coli* biorefining applications. Microb Cell Fact.

[33] Balzer GJ, Thakker C, Bennett GN, San KY (2013). Metabolic engineering of Escherichia coli to minimize byproduct formate and improving succinate productivity through increasing NADH availability by heterologous expression of NAD(+)-dependent formate dehydrogenase. Metab Eng.

[34] Litsanov B, Brocker M, Bott M (2012). Toward homosuccinate fermentation: metabolic engineering of Corynebacterium glutamicum for anaerobic production of succinate from glucose and formate. Appl Environ Microbiol.

[35] Piganeau M, Ghezraoui H, De Cian A, Guittat L, Tomishima M, Perrouault L, Rene O, Katibah G, Zhang L, Holmes M, Doyon Y, Concordet JP, Giovannangeli C, Jasin M, Brunet E (2013). Cancer translocations in human cells induced by zinc finger and TALE nucleases. Genome Res.

[36] Mali P, Yang LH, Esvelt KM, Aach J, Guell M, DiCarlo JE, Norville JE, Church GM (2013). RNA-guided human genome engineering via Cas9. Science.

[37] Kim Y, Kweon J, Kim A, Chon JK, Yoo JY, Kim HJ, Kim S, Lee C, Jeong E, Chung E, Kim D, Lee MS, Go EM, Song HJ, Kim H, Cho N, Bang D, Kim JS (2013). A library of TAL effector nucleases spanning the human genome. Nat Biotechnol.

[38] Dicarlo JE, Norville JE, Mali P, Rios X, Aach J, Church GM (2013). Genome engineering in *Saccharomyces cerevisiae* using CRISPR-Cas systems. Nucleic Acids Res.

[39] Cong L, Ran FA, Cox D, Lin SL, Barretto R, Habib N, Hsu PD, Wu XB, Jiang WY, Marraffini LA, Zhang F (2013). Multiplex genome engineering using CRISPR/Cas systems. Science.

[40] Gietz RD, Schiestl RH, Willems AR, Woods RA (1995). Studies on the transformation of intact yeast cells by the LiAc/SS-DNA/PEG procedure. Yeast.

[41] Higuchi R, Krummel B, Saiki RK (1988). A general method of in vitro preparation and specific mutagenesis of DNA fragments: study of protein and DNA interactions. Nucleic Acids Res.

[42] Shao Z, Zhao H, Zhao H (2009). DNA assembler, an in vivo genetic method for rapid construction of biochemical pathways. Nucleic Acids Res.

[43] Liang J, Ning JC, Zhao H (2013). Coordinated induction of multi-gene pathways in *Saccharomyces cerevisiae*. Nucleic Acids Res.

[44] Teste MA, Duquenne M, Francois JM, Parrou JL (2009). Validation of reference genes for quantitative expression analysis by real-time RT-PCR in *Saccharomyces cerevisiae*. BMC Mol Biol.

