# Supplementary material for: Exploiting Issatchenkia orientalis SD108 for succinic acid production
Source: Microb Cell Fact. 2014 Aug 27;13:121. doi: 10.1186/s12934-014-0121-4 (PMC4244060; doi:10.1186/s12934-014-0121-4)
Supplement: Additional file 1: — Supplementary Data. [file 12934_2014_121_MOESM1_ESM.docx]

**Supplementary Data**

**Isolation and Identification of SD108**

SD108 was isolated by inoculating a rotting bagasse sample into liquid YP medium (1% yeast extract and 2% peptone), unbuffered at pH 5, containing 50 g/L glucose and appropriate antibiotics (30 mg/L of chloramphenicol and 150 mg/L of ampicillin) in shake flasks fitted with water filled bubblers (gas traps) and incubating for 48 hours (30 °C, 100 rpm). The enriched cultures were then plated on either YP-2% xylose plates supplemented with appropriate antibiotics or on synthetic complete (SC) medium with 2% xylose and appropriate antibiotics supplemented and incubated aerobically at 30 °C. Colonies were obtained from the same plates and then the species were identified by DNA sequencing of a portion of the gene that encodes the D1/D2 domain of the large subunit of rRNA. Yeast SD108 genomic DNA was used as template to generate a PCR product with primers SD123 and SD124 (Table S1). When sequenced with primers SD124 and SD125 (Table S1), this PCR product gave a 599 base pair contiguous sequence which was 100% identical with the homologous sequence from *Issatchenkia orientalis* (also known as *Pichia kudriavzevii*) strain ATCC 24210 rDNA (Genbank accession number EF550222.1).

**Genetic Characterization - Genome Sequence, Size, and Ploidy**

In total, approximately 1.5 Gb of genomic information was used to generate 5326 contiguous sequences (≥ 100 bp). Of these contigs, 3,001 were incorporated into 364 scaffolds that sum up to a total sequence length of 11,409,147 bp. A summary of the sequencing approach and the *de novo* assembly is shown in Table S2. The gene prediction program, GeneMark-ES (http://exon.gatech.edu/), was used to predict genes in the above assembled scaffolds and contigs that were not incorporated into scaffolds. The program predicted 5,093 genes. Sequences of these genes were extracted from the assembled scaffolds and contigs based on the coordinates in the GTF file generated by GeneMark using several custom Perl and AWK scripts. These predicted gene sequences were aligned against two database sequences of *Pichia stipitis* and *S. cerevisiae* to extract annotation of homologous genes. Among the 5,093 predicted protein-coding sequences, more than 85% had hits in the two reference genomes. The percentages of protein-coding sequences which share above 50% homology with those from *P. stipitis* and *S. cerevisiae* are 45.3% and 40.3%, respectively. The predicted protein-coding regions cover 62.0% (7,839,088 bp) of the genome sequence, and their average length is 1539 bp. A schematic of predicted genes in *I. orientalis* SD108, related to carbohydrates utilization, glycolysis, pentose phosphate pathway, pyruvate metabolism, TCA cycle and glyoxylate shunt pathway is provided (Fig. S3).

A protocol based on chromosomal DNA staining and flow cytometry was subsequently applied to determine the genome size and ploidy [[1](#_ENREF_1), [2](#_ENREF_2)]. Haploid and diploid strains of *S. cerevisiae*, both of which are isogenic to the previously sequenced *S. cerevisiae* strain S288C with a haploid genome of 12,068 kilobases, were used to set up a calibration curve [[3](#_ENREF_3)]. These strains were grown to their exponential phases, diluted to the same density, and subjected to fixation and staining. Flow cytometry analysis revealed three major peaks (Fig. S4A), corresponding to 1*n*, *2n*, and 4*n* DNA contents, where the mean green fluorescence intensity of each peak was proportional to the amount of DNA (Mb) in each cell for the corresponding cell types (Fig. S4B, R^2^ > 0.99). 1*n* and 2*n* corresponded to the haploid and the diploid at *G0/G1* phases, respectively. 4*n* corresponded to the diploid at its *S* phase. The genome size of SD108 was estimated based on the R1 subpopulation (at *G0/G1* phase) of its cell cycle profile (Fig. S4C), which was calculated to be 20.1±2.6 Mb (Table S3). Since the draft genome has a total sequence length of 11,409,147 bp, SD108 was concluded to be a diploid. The genome length of SD108 is consistent with the recently published genome sequence of *P. kudriavzevii* (a synonymous name of *I. orientalis*) M12 [[4](#_ENREF_4)] which is approximately 10.4 Mb.

**DNA Transformation of SD108 and its Derived Strains**

A single colony grown on a YPAD plate was inoculated into 3 mL of YPAD medium in a 15 mL round-bottom Falcon tube and grown overnight until saturation (30 °C, 250 rpm). Approximately 150 µL of the stationary-phase cells were transferred into 20 mL of YPAD media. The initial OD_600_ was 0.2, and the cells were continuously grown for 4 h (30 °C, 250 rpm). Cells were collected by centrifugation, washed twice with deionized water and resuspended in 360 µL of transformation mixture consisting of 240 µL of 50% w/v PEG3350, 36 µl of 1 M lithium acetate, 50 µL of 2 mg/mL deoxyribonucleic acid from salmon testes (SS-DNA) that was boiled at 100 °C for 5 min and quickly chilled on ice, DNA (plasmid, linearized plasmid DNA or PCR products with the concentration in a range from 1 µg/µL to 5 µg/µL), and deionized water. After mixing thoroughly, the suspension was subjected to heat shock for various lengths of time (40-120 min) at 42 °C. Cells were collected by centrifugation, resuspended with 1 mL of YPAD and recovered for 2 h (30 °C, 250 rpm), after which cells were centrifuged, washed twice with 1 M sorbitol and then spread on SC-URA plates.

**Optimization of the DNA Transformation Protocol for SD108**

A Ura3 expression cassette was PCR amplified from genomic DNA of SD108 using primer Ura3-p-up and Ura3-t-dn (Table S1). Different amounts of the cassette were transformed into strain IoΔura3, the uracil auxotroph strain of SD108, using different heat shock times (Table S4). Colonies appeared on the SC-URA plates were counted for calculation of the transformation efficiency. Heat shock at 42 °C for 60 min gave the highest transformation efficiency (~10^3^), while increasing the DNA amount did not change the transformation efficiency significantly (Table S4). The optimal DNA transformation condition (1 µg DNA and heat shock at 42 °C for 60 min) was adopted for subsequent DNA transformation experiments.

**Identification of an Endogenous Autonomously Replicating Sequence (ARS)**

Plasmid construction was performed using the In-fusion HD Cloning Kit (Clontech Laboratories, Inc, USA). The zeocin expression cassette was amplified from the template plasmid pPICZαA using primers Zeocin-up and Zeocin-dn (Table 1 and S1), and subsequently cloned into plasmid pPK2 linearized by *Cla*I, yielding plasmid pPK2-zeocin. The plasmid pXZ1 was constructed by self-ligation of plasmid pPK2-zeocin linearized by *Bsm*BI digestion.

In a 100 µL reaction, 4 U *Sau*3AI enzyme was added to digest 10 µg genomic DNA of SD108 for 30 min at 37 °C. After inactivation at 65 °C for 20 min, the digestion mixture was purified using the QIAquick PCR Purification and Gel Extraction Kits (Qiagen, Valencia, CA). The plasmid pXZ1 was linearized and dephosphorylated by *Bam*HI and Thermosensitive Alkaline Phosphatase (FastAP) digestion, respectively. Ligation was performed by T4 ligase with 300 ng genomic DNA fragments and 100 ng linearized plasmid pXZ1 in 10 µL total volume. A total of 100 μL NEB 5α Electrocompetent *E. coli* were transformed with 1 µL of the ligation product by electroporation. The *E. coli* cells were immediately transferred into 1 mL SOC medium. After recovery for 1 h at 37 °C, all the cells were used to inoculate 25 mL LB medium plus 100 μg/mL ampicillin (Amp) and allowed to grow until saturation. One hundredth of the cells was plated on an LB plate containing Amp to estimate the transformation efficiency. A library size of 3.55×10^7^ CFU (µg^-1^ DNA) was obtained, while the control reaction with only the linearized plasmid gave 2.15×10^6^ CFU (µg^-1^ DNA) transformants. The plasmid library was isolated from the overnight *E. coli* culture, and 1 µg of DNA was transformed into strain IoΔura3 using the heat shock protocol described above. Cells were spread on fresh YPAD medium containing 50 μg/mL zeocin. Colonies grown on the YPAD plate plus zeocin were inoculated into the YPAD liquid medium containing 50 μg/mL zeocin for 16-20 h (30 °C, 250 rpm). Yeast plasmids were isolated and transferred into *E. coli* BW25141. The plasmid was purified from *E. coli* transformants and transformed in strain IoΔura3 for verification. The positive plasmid pXZ2 was subsequently sequenced with primer pXZ1-ARS-up (Table S1), and a functional endogenous ARS of SD108 was isolated (Fig. S6).

**Assembly of the Reductive TCA Pathway for Succinic Acid Production**

To construct plasmid pRS415-A, the *ura3* upstream homology arm and the downstream homology arm, *pdc1* terminator and the *tef1a* terminator were amplified from the genomic DNA of SD108 using primers A-ura3p-up/A-ura3-dn, A-PDCt-up/A-PDCt-dn, and A-TEF1at-up/A-TEF1at-dn, respectively (Table S1). A total of 0.2 pmol of each cassette was mixed together with 0.2 pmol of plasmid pRS415 linearized by *Hind*III and *Pst*I, and electroporated into *S. cerevisiae* BY4741 following the protocol described elsewhere [[5](#_ENREF_5)]. Colonies grown on SC-LEU plates were inoculated into the SC-LEU liquid medium for 36 h (30 °C, 250 rpm). Yeast plasmids were isolated using Zymoprep II yeast plasmid isolation kit (Zymo Research, Irvine, CA) and transferred into *E. coli* BW25141. *E. coli* plasmids were isolated using the plasmid DNA miniprep kit from Qiagen (Valencia, CA) and subjected to restriction digestion. Correct restriction digestion patterns were verified by DNA electrophoresis. Plasmid pRS415-B, pRS415-C and pRS415-D were prepared by following the construction strategy of plasmid pRS415-A, and primers are summarized in Table S1. Except for the *frd* gene, which was amplified from plasmid pFRD, all the genes, promoters and terminators were amplified from genomic DNA of SD108.

The DNA assembler method [[5](#_ENREF_5)] was used to construct plasmid pRS415-E. The *pyc* expression cassette was obtained from plasmid pRS415-B linearized with *Not*I, *Pme*I, *Adh*I and *Bsr*GI digestion. The *mdh* and *fumr* expression cassette was obtained from plasmid pRS415-C linearized with *Apa*I, *Ahd*I and *Sfo*I digestion. The *frd* expression cassette was obtained from plasmid pRS415-D linearized with *Eco*RI, *Ahd*I and *Sal*I digestion. To reduce the risk of point mutations introduced by PCR, three independent experiments were carried out in parallel. A total of 0.2 pmol of each set of expression cassettes, encoding *pyc*, *mdh*, *fumr* and *frg*, were mixed together with 0.2 pmol of plasmid pRS415-A linearized by *Not*I and *Spe*I digestion, and electroporated into *S. cerevisiae* BY4741. Yeast plasmids were isolated and transferred into *E. coli* BW25141. *E. coli* plasmids were isolated and subjected to restriction digestion. Correct restriction digestion patterns were verified by DNA electrophoresis. A cartoon depiction of the assembled four-gene expression cassette is shown in Fig. S7. Six fragments of the assembled reductive TCA pathway were obtained from six pRS415-E plasmids linearized by *Mlu*I digestion. After ethanol precipitation and resuspension in 10 µl of deionized water, 1 µg of each of these six fragments were transformed into strain IoΔura3 using heat shock. Ten engineered strains were generated from integrating five cassettes of the assembled reductive TCA pathway into the *ura3* (gene *JL09_g1026*) locus of strain IoΔura3, while no colonies were obtained from integration of the sixth cassette.

Table S1. Primers used in this study.

| **Primer name** | **Sequence (5’-3’)** | **Description** |
| --- | --- | --- |
| SD123 | GGAAGTAAAAGTCGTAACAAGG | Forward primer for 26S RNA |
| SD124 | CGCCAGTTCTGCTTACC | Sequencing and reverse primer for 26S RNA |
| SD125 | GCATATCAATAAGCGGAGGAAAAG | Sequencing primer for 26S RNA |
| Ura-p-up | Aaacagggaaggttgacatt | Forward primer for *ura3* promoter |
| Ura-p-dn | atttaaactagtgtacatgtatcaaatctttgtgtaagaaccttgacaaacaaactactt | Reverse primer for *ura3* promoter |
| Ura-t-up | agatcttcaacgctttaataaagtagtttgtttgtcaaggttcttacacaaagatttgat | Forward primer for *ura3* terminator |
| Ura-t-dn | aacacttagaatacgcggaa | Reverse primer for *ura3* terminator |
| pXZ1-ARS-up | aagttgggtaacgccagggt | Sequencing primers for plasmid pXZ2 |
| Zeocin-up | aaaacacatatcgacatcatcccacacaccatagcttcaa | Forward In-fusion primer for zeocin expression cassette |
| Zeocin-dn | CCTCGTACGAGAAGTCATCGAGCTTGCAAATTAAAGCCTT | Reverse In-fusion primer for zeocin expression cassette |
| A-ura3p-up | attgggtaccgggccccccctcgaggtcgacggtatcgatacgcgtaaacagggaaggttgacatt | Forward primer of *ura3* promoter for construction of plasmid pRS415-A |
| A-ura3-dn | TAGTAATTCATTTTAATGTTCATTTTACATTCAGATGTCATCATTTAAATCTGTTTAAAT | Reverse primer of gene *ura3* for construction of plasmid pRS415-A |
| A-PDCt-up | acaagctggttggaatgcttatttaaacagatttaaatgatgacatctgaatgtaaaatg | Forward primer of *pdc* terminator for construction of plasmid pRS415-A |
| A-PDCt-dn | AGAGTAAAGAAACTTCTCGTTCATCTTATTCTTTAGCCAAGTTTAAACTTTTTATTATAAAATTATAT | Reverse primer of *pdc* terminator for construction of plasmid pRS415-A |
| A-TEF1at-up | gatatgtaattaagaataatatataattttataataaaaagtttaaacttggctaaagaataagatga | Forward primer of *tef1a* terminator for construction of plasmid pRS415-A |
| A-TEF1at-dn | GCTGGAGCTCCACCGCGGTGGCGGCCGCTCTAGAACTAGTCGATCGGTATAGCCATATAGTTTAAT | Reverse primer of *tef1a* terminator for construction of plasmid pRS415-A |
| B-TEF1at-up | attgggtaccgggccccccctcgaggtcgacggtatcgatgtttaaacttggctaaagaataagatga | Forward primer of *tef1a* terminator for construction of plasmid pRS415-B |
| B-TEF1at-dn | TTTGCTTGTGAAAATTGCCAAAGATGAAGCGCCAGCTTAAGTATAGCCATATAGTTTAAT | Reverse primer of *tef1a* terminator for construction of plasmid pRS415-B |
| B-PYC-up | gttataaaaagtataaaggaattaaactatatggctatacttaagctggcgcttcatctt | Forward primer of gene *pyc* for construction of plasmid pRS415-B |
| B-PYC-dn | TACTACTACTACTATTACTACCACCCCCAACACAAACACAATGTCAACTGTGGAAGATCA | Reverse primer of gene *pyc* for construction of plasmid pRS415-B |
| B-FBA1p-up | tcaatttatgtaaggaggagtgatcttccacagttgacattgtgtttgtgttgggggtgg | Forward primer of *fba1* promoter for construction of plasmid pRS415-B |
| B-FBA1p-dn | GCTGGAGCTCCACCGCGGTGGCGGCCGCTCTAGAACTAGTCGATCGATTGTATGTGTATTGTATTA | Reverse primer of *fba1* promoter for construction of plasmid pRS415-B |
| C-FBA1p-up | attgggtaccgggccccccctcgaggtcgacggtatcgatgggccctgtgtttgtgttgggggtgg | Forward primer of *fba1* promoter for construction of plasmid pRS415-C |
| C-FBA1p-dn | TGCTTCACAGAGGGTGAAACAGTTTTCATGATGTTTCAAACGATCGATTGTATGTGTATTGTATTA | Reverse primer of *fba1* promoter for construction of plasmid pRS415-C |
| C-TEF1ap-up | cacttaagacaatacacacttaatacaatacacatacaatcgatcgtttgaaacatcatgaaaact | Forward primer of *tef1a* promoter for construction of plasmid pRS415-C |
| C-TEF1ap-dn | CAATTCCACCGGCAGCGCCTAAAATAGTCACCTTGACCATTGTGATATATAAGTTAGATT | Reverse primer of *tef1a* promoter for construction of plasmid pRS415-C |
| C-MDH-up | actttttccttcaacagacaaatctaacttatatatcacaatggtcaaggtgactatttt | Forward primer of gene *mdh* for construction of plasmid pRS415-C |
| C-MDH-dn | ATCTATATACATACACAACCAGCTGATTCAAGAGAGGCTTTTAGCCATGGACAAAATTGA | Reverse primer of gene *mdh* for construction of plasmid pRS415-C |
| C-Trp3t-up | agttgacgttaataaaggcttcaattttgtccatggctaaaagcctctcttgaatcagct | Forward primer of *trp3* terminator for construction of plasmid pRS415-C |
| C-Trp3t-dn | TCTAACCTAAGGACTTAAATATTTGTACAAACATGTTCCACGATCGTGTTCAAATGCTTGTCTGGT | Reverse primer of *trp3* terminator for construction of plasmid pRS415-C |
| C-ENO2t-up | ggctagttctgcgcctcttcaccagacaagcatttgaacacgatcgtggaacatgtttgtacaaat | Forward primer of *eno2* terminator for construction of plasmid pRS415-C |
| C-ENO2t-dn | ATGGGTTAGACCAGAAAACATGATTGGTCCAAAGGATTAGATCTAACTAATGCTTTTACT | Reverse primer of *eno2* terminator for construction of plasmid pRS415-C |
| C-FUMR-up | aaaaattgtactagatatttagtaaaagcattagttagatctaatcctttggaccaatca | Forward primer of gene *fumr* for construction of plasmid pRS415-C |
| C-FUMR-dn | TAACACAACAAAAACAAAAACAACGACAACAACAACAACAATGTTCTCAACTACCTCAAT | Reverse primer of gene *fumr* for construction of plasmid pRS415-C |
| C-PGK1p-up | ctttttcgattctttttgcaattgaggtagttgagaacattgttgttgttgttgtcgttg | Forward primer of *pgk1* promoter for construction of plasmid pRS415-C |
| C-PGK1p-dn | GCTGGAGCTCCACCGCGGTGGCGGCCGCTCTAGAACTAGTGGGCCCATGTGCTGAATGCCATGGCA | Reverse primer of *pgk1* promoter for construction of plasmid pRS415-C |
| D-PGK1p-up | attgggtaccgggccccccctcgaggtcgacggtatcgatacgcgttgttgttgttgttgtcgttg | Forward primer of *pgk1* promoter for construction of plasmid pRS415-D |
| D-PGK1p-dn | TAAAAAATAGACATACCCCTTTTGGATCAGGTTAAATCAACGATCGATGTGCTGAATGCCATGGCA | Reverse primer of *pgk1* promoter for construction of plasmid pRS415-D |
| D-TDH3p-up | cccaatgtaggactctgagttgccatggcattcagcacatcgatcgttgatttaacctgatccaaa | Forward primer of *tdh3* promoter for construction of plasmid pRS415-D |
| D-TDH3p-dn | CAACGGCAACTATGCTCGCAGATGATCTACCATCAACCATTTTTTGTAATTGTGTTTGTT | Reverse primer of *tdh3* promoter for construction of plasmid pRS415-D |
| D-FRD-up | caaaacacacaaaacacacaaacaaacacaattacaaaaaatggttgatggtagatcatc | Forward primer of gene *frd* for construction of plasmid pRS415-D |
| D-FRD-dn | GTATTTTATATTAAATTCATTACGTTGATCTATGTTTGATTTATGACCCACTTGGTTCAG | Reverse primer of gene *frd* for construction of plasmid pRS415-D |
| D-PGK1t-up | ggtcagaacggttgacgaaactgaaccaagtgggtcataaatcaaacatagatcaacgta | Forward primer of *pgk1* terminator for construction of plasmid pRS415-D |
| D-PGK1t-dn | ATTTAAACTAGTGTACATGTATCAAATCTTTGTGTAAGAAGTTTAAACTATGATAATAATAATAATAA | Reverse primer of *pgk1* terminator for construction of plasmid pRS415-D |
| D-ura3t-up | tattattattattattattattattattattattatcatagtttaaacttcttacacaaagatttgat | Forward primer of *ura3* terminator for construction of plasmid pRS415-D |
| D-ura3t-dn | GCTGGAGCTCCACCGCGGTGGCGGCCGCTCTAGAACTAGTACGCGTAACACTTAGAATACGCGGAA | Reverse primer of *ura3* terminator for construction of plasmid pRS415-D |
| q-alg9-up | GCCATTAGAAGCTATGCGTATTTG | Forward qPCR primer for gene *alg9* |
| q-alg9-dn | GGCCAAAACCAAACGAACAG | Reverse qPCR primer for gene *alg9* |
| q-pyc-up | GGCATTGCTAACTCATCC | Forward qPCR primer for gene *pyc* |
| q-pyc-dn | TTTGGGCTCTATTCTTTG | Reverse qPCR primer for gene *pyc* |
| q-mdh-up | AGAACTAAAGCGTTTGAATG | Forward qPCR primer for gene *mdh* |
| q-mdh-dn | ATAGAGTCGCCCGAATGG | Reverse qPCR primer for gene *mdh* |
| q-fumr-up | TGAAATCTTGGGTGGTGA | Forward qPCR primer for gene *fumr* |
| q-fumr-dn | ATGGGATGAAACTTCTGTAA | Reverse qPCR primer for gene *fumr* |
| q-frd-up | GCATAGTTGCCGTTGACC | Forward qPCR primer for gene *frd* |
| q-frd-dn | GCCCAGATGTTGCGTATT | Reverse qPCR primer for gene *frd* |
| q-tdh3-up | ggtagaactgcttccggtaac | Forward qPCR primer for gene *tdh3* |
| q-tdh3-dn | aacagagacatcgacagttgg | Reverse qPCR primer for gene *tdh3* |
| q-tef1a-up | ttaccgttattgatgctccagg | Forward qPCR primer for gene *tef1a* |
| q-tef1a-dn | agcgtgttctctagtttgacc | Reverse qPCR primer for gene *tef1a* |
| q-fba1-up | acccaatatgcatacttgactgg | Forward qPCR primer for gene *fba1* |
| q-fba1-dn | ttttcaccttctctgacccac | Reverse qPCR primer for gene *fba1* |
| q-pgk1-up | ggtaaggtcaaggcagataagg | Forward qPCR primer for gene *pgk1* |
| q-pgk1-dn | ctctggcaattcgaaaccaac | Reverse qPCR primer for gene *pgk1* |

Table S2. Genome sequencing and assembly results of *I. orientalis* SD108.

| **Features of the genome sequencing** | **Result** |
| --- | --- |
| Number of sequence reads | 1,601,359 for shotgun library;  1,110,604 for pair-end library |
| Number of sequenced bases | 1,014,145,406 for shotgun library;  446,561,488 for pair-end library |
| Size of assembled sequence (bp) | 12,642,999 |
| Number of all contigs | 5,326 |
| Largest contig (bp) | 173,329 |
| N50 contig size (bp) | 5,124 |
| Number of scaffolds | 364 |
| Number of contigs in scaffolds | 3,001 |
| Size of assembled scaffolds (bp) | 11,409,147 |
| N50 scaffold size (bp) | 160,993 |

Table S3. Genome size (Mb) of SD108 estimated by flow cytometry of SYBR Green I-stained cells.

| **Microorganism** | **MFI ± SD^a^** | **Genome size ± SD (Mb)** |
| --- | --- | --- |
| *S. cerevisiae* |  |  |
| BY4741 | 259.0 ± 45.8 | 12.1 ± 2.1^b^ |
| PRT238 | 488.1 ± 32.1 | 24.2 ± 1.6^b^ |
| *I. orientalis* |  |  |
| SD108 | 414.1 ± 42.6 | 20.1 ± 2.6 |

^a^The mean fluorescence intensity (MFI) of cells in G0/G1 phases or R1 subpopulation.

^b^The theoretical genome sizes of *S. cerevisiae* haploid strain and diploid strain at its S phase [[3](#_ENREF_3)].

Table S4. DNA transformation efficiency of the IoΔura3 strain. CFU = colony forming unit.

| Amount of DNA (µg) | 1 | | | | | | 2 | 5 |
| --- | --- | --- | --- | --- | --- | --- | --- | --- |
| Incubation time at 42 ºC (min) | 40 | 60 | 75 | 90 | 105 | 120 | 90 | 90 |
| CFU (µg^-1^ DNA) | 218 | 996 | 460 | 55 | 6 | 0 | 36 | 62 |

Table S5. Candidate genes related to acid tolerance in *I. orientalis* SD108.

| **Gene** | **Predicted protein function** | **Reference** |
| --- | --- | --- |
| *JL09_g597* | Plasma membrane H^+^-ATPase | [[6](#_ENREF_6)] |
| *JL09_g945* | Plasma membrane transporter of the major facilitator superfamily | [[7](#_ENREF_7)] |
| *JL09_g2976, JL09_g2752, JL09_g3122, JL09_g229, JL09_g2525, JL09_g1218, JL09_g1943, JL09_g5009, JL09_g3297, JL09_g4488, JL09_g2983* and *JL09_g2275* | Vacuolar H^+^- ATPase | [[8](#_ENREF_8)] |
| *JL09_g1177, JL09_g2415, JL09_g4516, JL09_g2533* and *JL09_g3253* | Plasma membrane ATP-binding cassette (ABC) transporter | [[8-10](#_ENREF_8)] |
| *JL09_g1637, JL09_g1636, JL09_g373 and JL09_g1946* | Polyamine transporter of the major facilitator superfamily | [[11](#_ENREF_11)] |
| *JL09_g2035* | Transcriptional activator involved in adaptation to weak acid stress | [[11](#_ENREF_11), [12](#_ENREF_12)] |
| *JL09_g5025* and *JL09_g5029* | Glycosylphosphatidylinositol-anchored cell wall protein involved in weak acid resistance | [[13](#_ENREF_13)] |
| *JL09_g4823* and *JL09_g4820* | C-8 sterol isomerase |  |
| *JL09_g3074* | C-5 sterol desaturase | [[14](#_ENREF_14)] |
| *JL09_g1927* | C-24(28) sterol reductase | [[14](#_ENREF_14)] |
| *JL09_g1130* | C-14 sterol reductase | [[14](#_ENREF_14)] |
| *JL09_g3258* | Oligomeric mitochondrial matrix chaperone | [[14](#_ENREF_14)] |
| *JL09_g3380* | Disaggregase | [[14](#_ENREF_14)] |
| *JL09_g1608* and *JL09_g3105* | Co-chaperone that stimulates HSP70 protein Ssc1p ATPase activity | [[14](#_ENREF_14)] |
| *JL09_g4596* | Alkaline dihydroceramidase | [[14](#_ENREF_14)] |
| *JL09_g1080* and *JL09_g823* | Phosphatidylinositol transfer protein | [[14](#_ENREF_14)] |
| *JL09_g2118, JL09_g3165, JL09_g2269, JL09_g3191, JL09_g1380, JL09_g2277, JL09_g1491, JL09_g202, JL09_g2141, JL09_g2530, JL09_g5071, JL09_g4970, JL09_g2187, JL09_g1697, JL09_g3599, JL09_g914, JL09_g1538,* and *JL09_g2998* | Putative plasma membrane sensor | [[14](#_ENREF_14)] |
| *JL09_g4045* | Regulates transcription of ribosomal protein and biogenesis genes | [[14](#_ENREF_14)] |
| *JL09_g48* | Transcription factor that is activated by a MAPK signaling cascade | [[14](#_ENREF_14)] |
| *JL09_g3139* | Nuclear protein that negatively regulates pseudohyphal differentiation | [[14](#_ENREF_14)] |
| *JL09_g1867* and *JL09_g2691* | Transcriptional activator activated in stress conditions | [[8](#_ENREF_8), [14](#_ENREF_14), [15](#_ENREF_15)] |
| *JL09_g2418* and *JL09_g340* | Cys2, His2 zinc-finger transcriptional repressor | [[14](#_ENREF_14)] |
| *JL09_g1975, JL09_g238* and *JL09_g4199* | Mitochondrial malate dehydrogenase | [[16](#_ENREF_16)] |
| *JL09_g3381* | Aconitase | [[16](#_ENREF_16)] |
| *JL09_g935* | Citrate synthase | [[16](#_ENREF_16)] |
| *JL09_g2435* | Subunit of mitochondrial NAD(+)-dependent isocitrate dehydrogenase | [[16](#_ENREF_16)] |
| *JL09_g3584* | Dihydrolipoamide dehydrogenase | [[16](#_ENREF_16)] |
| *JL09_g2373* | Alpha subunit of the F1 sector of mitochondrial F1F0 synthase | [[16](#_ENREF_16)] |
| *JL09_g2239* | Beta subunit of the F1 sector of mitochondrial F1F0 synthase | [[16](#_ENREF_16)] |
| *JL09_g422* | Mitochondrial manganese superoxide dismutase | [[16](#_ENREF_16)] |
| *JL09_g2077* and *JL09_g2078* | Dihydroxyacetone kinase | [[16](#_ENREF_16)] |
| *JL09_g1629* | Mitochondrial porin (voltage-dependent anion channel) | [[16](#_ENREF_16)] |
| *JL09_g3148* | Ser/Thr kinase involved in transcription and stress response | [[17](#_ENREF_17)] |
| *JL09_g948* | Calmodulin-dependent protein kinase | [[18](#_ENREF_18)] |
| *JL09_g4447* and *JL09_g4742* | AMP-activated serine/threonine protein kinase | [[19](#_ENREF_19)] |
| *JL09_g1345* | Basic helix-loop-helix protein | [[19](#_ENREF_19)] |
| *JL09_g3061* | Transcriptional repressor | [[19](#_ENREF_19)] |
| *JL09_g1159* and *JL09_g291* | Transcription factor | [[19](#_ENREF_19)] |
| *JL09_g868* | Transcription cofactor | [[19](#_ENREF_19)] |
| *JL09_g2932* | Component of the Rpd3L histone deacetylase complex | [[19](#_ENREF_19)] |
| *JL09_g4123* | Transcription factor required for septum destruction after cytokinesis | [[19](#_ENREF_19)] |
| *JL09_g4648* | Basic leucine zipper transcription factor in ATF/CREB family | [[19](#_ENREF_19)] |
| *JL09_g3392* | bHLH/Zip transcription factor for retrograde and TOR pathways | [[19](#_ENREF_19)] |
| *JL09_g3982, JL09_g4465, JL09_g881, JL09_g4678* and *JL09_g4682* | Transcriptional activator for GABA-dependent induction of GABA gene | [[19](#_ENREF_19)] |
| *JL09_g178* and *JL09_g1541* | Transcriptional repressor that regulates hypoxic genes during normoxia | [[19](#_ENREF_19)] |
| *JL09_g4795* and *JL09_g4796* | Serine-rich protein that contains a bHLH DNA binding motif | [[19](#_ENREF_19)] |
| *JL09_g602* | Transcriptional activator of thiamine biosynthetic genes | [[19](#_ENREF_19)] |
| *JL09_g875, JL09_g3852* and *JL09_g877* | Zinc finger transcription factor | [[19](#_ENREF_19)] |

Figure S1. The growth curve of SD108 in SC medium containing 5 g/L of glucose or 5 g/L of fructose.


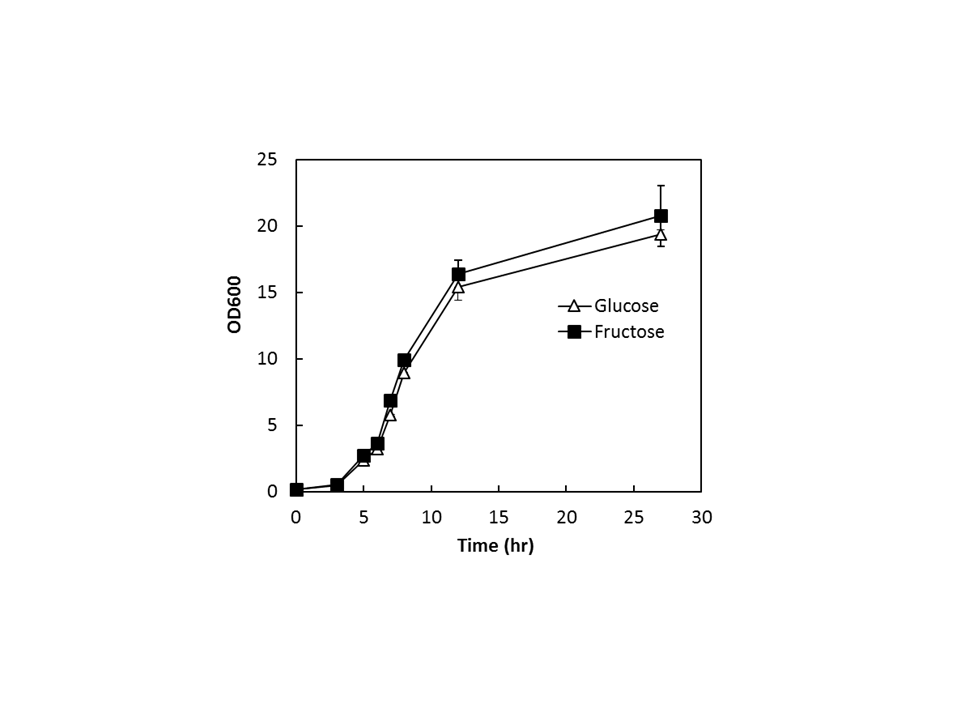


Figure S2. The pH change during measuring maximum specific growth rates of *I. orientalis* SD108 in SC medium at different starting pH values.


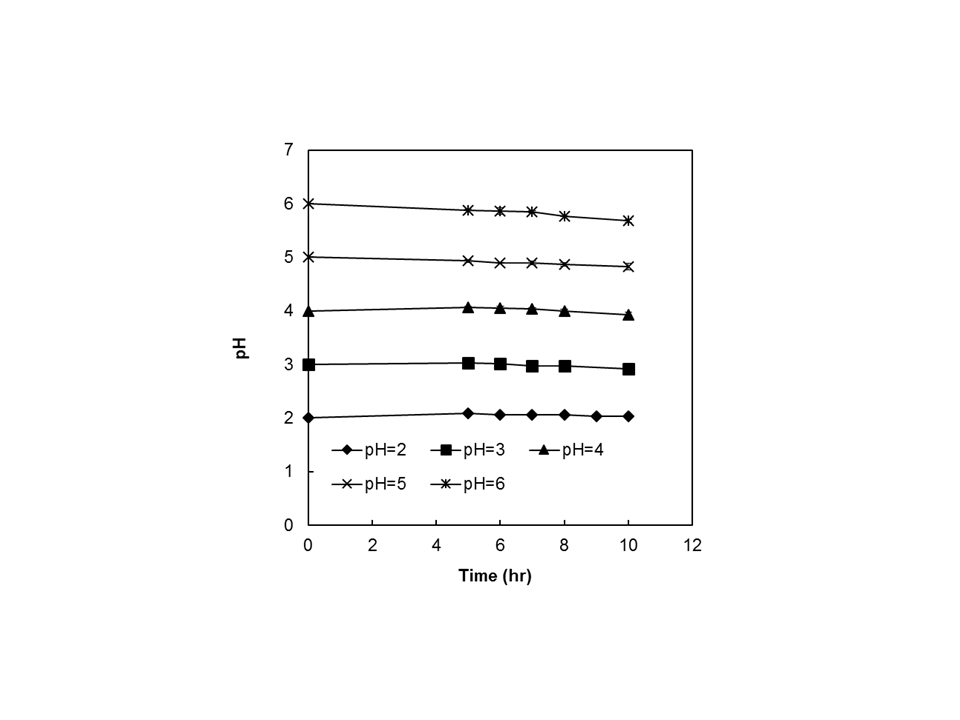


Figure S3. Schematic of predicted genes, related to carbohydrate utilization, glycolysis, pentose phosphate pathway, pyruvate metabolism, TCA cycle and glyoxylate shunt pathway, in *I. orientalis* SD108. Production of itaconic acid and adipic acid from intermediates of TCA cycle were depicted by dashed arrows. Abbreviations: G6P, glucose-6-phopsphate; F1P, fructose-1-phosphate; F6P, fructose-6-phosphate; FBP, fructose 1,6-bisphosphate; DHAP, dihydroxyacetone phosphate; GAP, glyceraldehyde-3-phosphate; 3PG, 3-phosphoglycerate; 2PG, 2-phosphoglycerate; PEP, phosphoenolpyruvate; 6PGL, 6-phospho-glucono-1,5-lactone; 6PG, 6-phosphogluconate; Ru5P, ribulose-5-phosphate; R5P, ribose-5-phosphate; X5P, xylulose-5-phosphate; S7P, seduheptulose-7-phosphate; E4P, erythrose-4-phosphate; OAA, oxaloacetate.


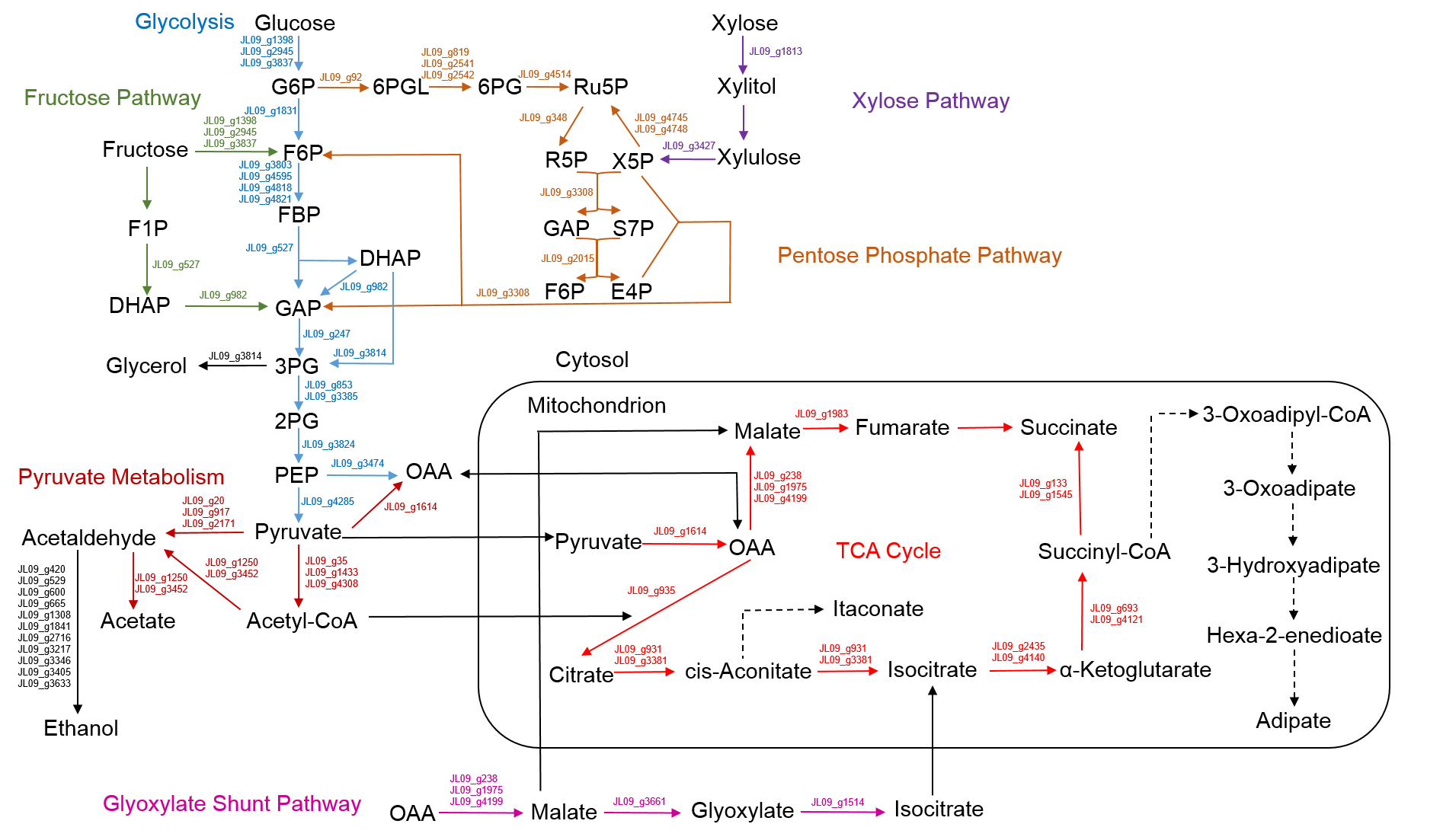


Figure S4. Genome size estimation of *I. orientalis* SD108 by flow cytometry. (A) The overlay histograms of the reference cells: the *S. cerevisiae* haploid BY4741 and the diploid PRT238; (B) The standard curve relating mean fluorescence intensity of the peaks 1*n*, 2*n* and 4*n* of *S. cerevisiae* strains in (A) and the theoretical amounts of DNA (in Mb) per cell; (C) The histogram of SD108 grown in the same condition.


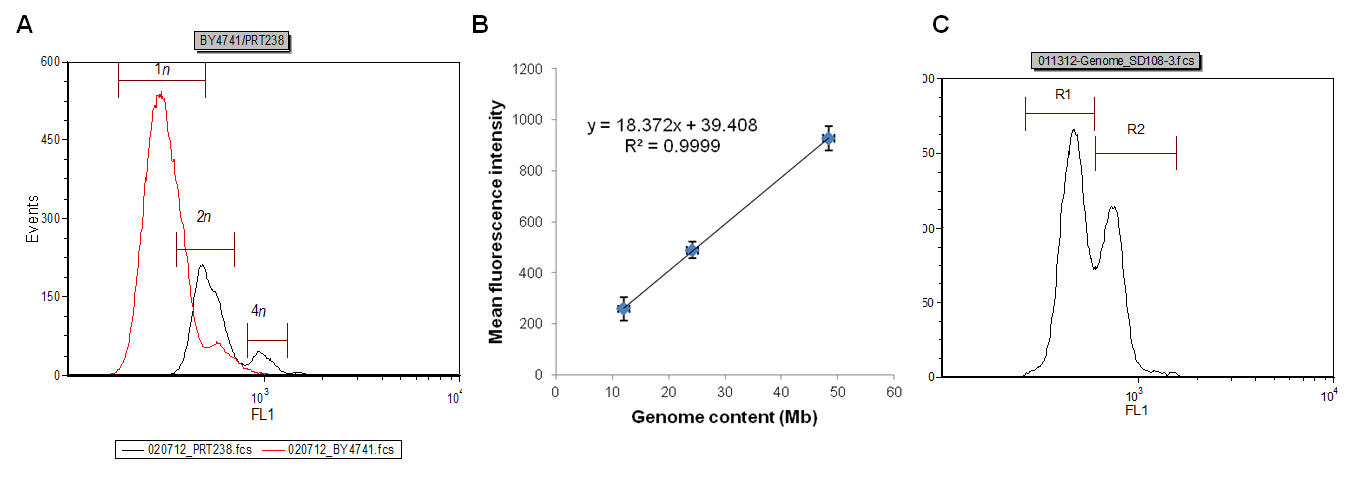


BY4741

PRT238

Figure S5. Deletion of *ura3* in *I. orientalis* SD108. (A) The double deletion mutants could only grow on SC+FOA (4 out of 8 positive clones are shown here); (B) Transforming a wild type *ura3* with homology arms to SD108 resulted in the recovery of ura3 function; (C) PCR analysis showed that for all the transformants, only one copy of ura3 was fixed (the ura3 deleted locus and the wide type locus amplified using primer Ura3-p-up and Ura3-t-dn are 822 bp and 1611 bp, respectively).

Figure S6. Sequencing result of the endogenous ARS (underlined) on pXZ2.

tcggtacccggggatccgaagatgaaccaaataggctattctttccagacttgtagaagtgataatgggccaaccaggttcaagtgtacaccattagaggatcctctagagtcgacctgcaggcatgcaagcttggcgtaatcatggtcatagctgtttcctgtgtgaaattgttatccgctcacaattccacacaacatacgagccggaagcataaagtgtaaagcctggggtgcctaatgagtgagctaactcacattaattgcgttgcgctcactgcccgctttccagtcgggaaacctgtcgtgccagctgcattaatgaatcggccaacgcgcggggagaggcggtttgcgtattgggcgctcttccgcttcctcgctcactgactcgctgcgctcggtcgttcggctgcggcgagcggtatcagctcactcaaaggcggtaatacggttatccacagaatcaggggataacgcaggaaagaacatgtgagcaaaaggccagcaaaaggccaggaaccgtaaaaaggccgcgttgctggcgtttttccataggctccgcccccctgacgagcatcacaaaaatcgacgctcaagtcagaggtggcgaaacccgacaggactataaagataccaggcgtttccccctggaagctccctcgtgcgctctcctgttccgaccctgccgcttaccggatacctgtccgcctttctcccttcgggaagcgtggcgctttctcatagct

Figure S7. Schematic of the engineered succinic acid operon.


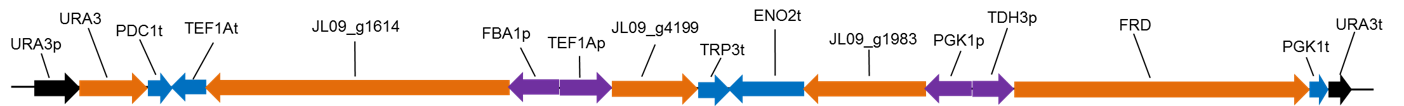


**References**

1. Almeida AJ, Martins M, Carmona JA, Cano LE, Restrepo A, Leao C, Rodrigues F: **New insights into the cell cycle profile of *Paracoccidioides brasiliensis*.** *Fungal Genet Biol* 2006, **43:**401-409.

2. Almeida AJ, Matute DR, Carmona JA, Martins M, Torres I, McEwen JG, Restrepo A, Leao C, Ludovico P, Rodrigues F: **Genome size and ploidy of *Paracoccidioides brasiliensis* reveals a haploid DNA content: flow cytometry and GP43 sequence analysis.** *Fungal Genet Biol* 2007, **44:**25-31.

3. Goffeau A, Barrell BG, Bussey H, Davis RW, Dujon B, Feldmann H, Galibert F, Hoheisel JD, Jacq C, Johnston M, Louis EJ, Mewes HW, Murakami Y, Philippsen P, Tettelin H, Oliver SG: **Life with 6000 genes.** *Science* 1996, **274:**546, 563-567.

4. Chan GF, Gan HM, Ling HL, Rashid NA: **Genome sequence of *Pichia kudriavzevii* M12, a potential producer of bioethanol and phytase.** *Eukaryot Cell* 2012, **11:**1300-1301.

5. Shao Z, Zhao H, Zhao H: **DNA assembler, an in vivo genetic method for rapid construction of biochemical pathways.** *Nucleic Acids Res* 2009, **37:**e16.

6. Holyoak CD, Bracey D, Piper PW, Kuchler K, Coote PJ: **The *Saccharomyces cerevisiae* weak-acid-inducible ABC transporter Pdr12 transports fluorescein and preservative anions from the cytosol by an energy-dependent mechanism.** *J Bacteriol* 1999, **181:**4644-4652.

7. Tenreiro S, Nunes PA, Viegas CA, Neves MS, Teixeira MC, Cabral MG, Sa-Correia I: **AQR1 gene (ORF YNL065w) encodes a plasma membrane transporter of the major facilitator superfamily that confers resistance to short-chain monocarboxylic acids and quinidine in *Saccharomyces cerevisiae*.** *Biochem Biophys Res Commun* 2002, **292:**741-748.

8. Schuller C, Mamnun YM, Mollapour M, Krapf G, Schuster M, Bauer BE, Piper PW, Kuchler K: **Global phenotypic analysis and transcriptional profiling defines the weak acid stress response regulon in *Saccharomyces cerevisiae*.** *Mol Biol Cell* 2004, **15:**706-720.

9. Hatzixanthis K, Mollapour M, Seymour I, Bauer BE, Krapf G, Schuller C, Kuchler K, Piper PW: **Moderately lipophilic carboxylate compounds are the selective inducers of the *Saccharomyces cerevisiae* Pdr12p ATP-binding cassette transporter.** *Yeast* 2003, **20:**575-585.

10. Balzi E, Wang M, Leterme S, Van Dyck L, Goffeau A: **PDR5, a novel yeast multidrug resistance conferring transporter controlled by the transcription regulator PDR1.** *J Biol Chem* 1994, **269:**2206-2214.

11. Fernandes AR, Mira NP, Vargas RC, Canelhas I, Sa-Correia I: ***Saccharomyces cerevisiae* adaptation to weak acids involves the transcription factor Haa1p and Haa1p-regulated genes.** *Biochem Biophys Res Commun* 2005, **337:**95-103.

12. Mira NP, Henriques SF, Keller G, Teixeira MC, Matos RG, Arraiano CM, Winge DR, Sa-Correia I: **Identification of a DNA-binding site for the transcription factor Haa1, required for *Saccharomyces cerevisiae* response to acetic acid stress.** *Nucleic Acids Res* 2011, **39:**6896-6907.

13. Simoes T, Teixeira MC, Fernandes AR, Sa-Correia I: **Adaptation of *Saccharomyces cerevisiae* to the herbicide 2,4-dichlorophenoxyacetic acid, mediated by Msn2p- and Msn4p-regulated genes: important role of SPI1.** *Appl Environ Microbiol* 2003, **69:**4019-4028.

14. Mira NP, Teixeira MC, Sa-Correia I: **Adaptive response and tolerance to weak acids in *Saccharomyces cerevisiae*: a genome-wide view.** *OMICS* 2010, **14:**525-540.

15. Hueso G, Aparicio-Sanchis R, Montesinos C, Lorenz S, Murguia JR, Serrano R: **A novel role for protein kinase Gcn2 in yeast tolerance to intracellular acid stress.** *Biochem J* 2012, **441:**255-264.

16. Guerreiro JF, Mira NP, Sa-Correia I: **Adaptive response to acetic acid in the highly resistant yeast species *Zygosaccharomyces bailii* revealed by quantitative proteomics.** *Proteomics* 2012, **12:**2303-2318.

17. Makrantoni V, Dennison P, Stark MJ, Coote PJ: **A novel role for the yeast protein kinase Dbf2p in vacuolar H+-ATPase function and sorbic acid stress tolerance.** *Microbiology* 2007, **153:**4016-4026.

18. Holyoak CD, Thompson S, Ortiz Calderon C, Hatzixanthis K, Bauer B, Kuchler K, Piper PW, Coote PJ: **Loss of Cmk1 Ca^2+^-calmodulin-dependent protein kinase in yeast results in constitutive weak organic acid resistance, associated with a post-transcriptional activation of the Pdr12 ATP-binding cassette transporter.** *Mol Microbiol* 2000, **37:**595-605.

19. Mira NP, Palma M, Guerreiro JF, Sa-Correia I: **Genome-wide identification of *Saccharomyces cerevisiae* genes required for tolerance to acetic acid.** *Microb Cell Fact* 2010, **9:**79.
